# MichiGAN: sampling from disentangled representations of single-cell data using generative adversarial networks

**DOI:** 10.1186/s13059-021-02373-4

**Published:** 2021-05-20

**Authors:** Hengshi Yu, Joshua D. Welch

**Affiliations:** 1 Department of Biostatistics, University of Michigan, Ann Arbor, USA; 2 Department of Computational Medicine and Bioinformatics, University of Michigan, Ann Arbor, USA; 3 Department of Computer Science and Engineering, University of Michigan, Ann Arbor, USA

**Keywords:** Cellular identity, Disentangled representations, Generative adversarial networks, Representation learning, Single-cell genomics

## Abstract

Deep generative models such as variational autoencoders (VAEs) and generative adversarial networks (GANs) generate and manipulate high-dimensional images. We systematically assess the complementary strengths and weaknesses of these models on single-cell gene expression data. We also develop MichiGAN, a novel neural network that combines the strengths of VAEs and GANs to sample from disentangled representations without sacrificing data generation quality. We learn disentangled representations of three large single-cell RNA-seq datasets and use MichiGAN to sample from these representations. MichiGAN allows us to manipulate semantically distinct aspects of cellular identity and predict single-cell gene expression response to drug treatment.

**Supplementary Information:**

The online version contains supplementary material available at (10.1186/s13059-021-02373-4).

## Introduction

Deep learning techniques have recently achieved remarkable successes, especially in vision and language applications [1, 2]. In particular, state-of-the-art deep generative models can generate realistic images or sentences from low-dimensional latent variables [3]. The generated images and text data are often nearly indistinguishable from real data, and data generating performance is rapidly improving [4, 5]. The two most widely types of deep generative models are variational autoencoders (VAEs) and generative adversarial networks (GANs). VAEs use a Bayesian approach to estimate the posterior distribution of a probabilistic encoder network, based on a combination of reconstruction error and the prior probability of the encoded distribution [6]. In contrast, the GAN framework consists of a two-player game between a generator network and a discriminator network [7]. GANs and VAEs possess complementary strengths and weaknesses: GANs generate much better samples than VAEs [8], but VAE training is much more stable and learns more useful disentangled latent representations [9]. GANs outperform VAEs in generating sharp image samples [7], while VAEs tend to generate blurry images [10]. GAN training is generally less stable than VAE training, but some recent derivations of GAN like Wasserstein GAN [11,13] significantly improve the stability of GAN training, which is particularly helpful for non-image data.

Achieving a property called disentanglement, in which each dimension of the latent representation controls a semantically distinct factor of variation, is a key focus of recent research on deep generative models [14,20]. Disentanglement is important for controlling data generation and generalizing to unseen latent variable combinations. For example, disentangled representations of image data allow prediction of intermediate images [21] and mixing images styles [22]. For reasons that are not fully understood, VAEs generally learn representations that are more disentangled than other approaches [23,28]. The state-of-the-art methods for learning disentangled representations capitalize on this advantage by employing modified VAE architectures that further improve disentanglement, including **-VAE, FactorVAE, and **-TCVAE [9, 29,31]. In contrast, the latent space of the traditional GAN is highly entangled. Some modified GAN architectures, such as InfoGAN [32], encourage disentanglement using purely unsupervised techniques, but these approaches still do not match the disentanglement performance of VAEs [33,40].

Disentanglement performance is usually quantitatively evaluated on standard image datasets with known ground truth factors of variation [41,44]. In addition, disentangled representations can be qualitatively assessed by performing traversals or linear arithmetic in the latent space and visually inspecting the resulting images [45,49].

Recently, molecular biology has seen the rapid growth of single-cell RNA-seq technologies that can measure the expression levels of all genes across thousands to millions of cells [50]. Like image data, for which deep generative models have proven so successful, single-cell RNA-seq datasets are large and high-dimensional. Thus, it seems likely that deep learning will be helpful for single-cell data. In particular, deep generative models hold great promise for distilling semantically distinct facets of cellular identity and predicting unseen cell states.

Several papers have already applied VAEs [51,61] and GANs [62] to single-cell data. A representative VAE method is scGen, which uses the same objective function as **-VAE [9]. The learned latent values in scGen are utilized for out-of-sample predictions by latent space arithmetic. The cscGAN paper adapts the Wasserstein GAN approach for single-cell data and shows that it can generate realistic gene expression profiles, proposing to use it for data augmentation.

Assessing disentanglement performance of models on single-cell data is more challenging than image data, because humans cannot intuitively understand the data by looking at it as with images. Previous approaches such as scGen have implicitly used the properties of disentangled representations [51], but disentanglement performance has not been rigorously assessed on single-cell data.

Here, we systematically assess the disentanglement and generation performance of deep generative models on single-cell RNA-seq data. We show that the complementary strengths and weaknesses of VAEs and GANs apply to single-cell data in a similar way as image data. We develop MichiGAN, a neural network that combines the strengths of VAEs and GANs to sample from disentangled representations without sacrificing data generation quality. We employ MichiGAN and other methods on simulated single-cell RNA-seq data [63, 64] and provide quantitative comparisons through several disentanglement metrics [29, 30]. We also learn disentangled representations of three real single-cell RNA-seq datasets [65,67] and show that the disentangled representations can control semantically distinct aspects of cellular identity and predict unseen combinations of cell states.

Our work builds upon that of Lotfollahi et al. [51], who showed that a simple VAE (which they called scGen) can predict single-cell perturbation responses. They also showed several specific biological contexts in which this type of approach is useful. First, they predicted the cell-type-specific gene expression changes induced by treating immune cells with lipopolysaccharide. Second, they predicted the cell-type-specific changes that occur when intestinal epithelial cells are infected by *Salmonella* or *Heligmosomoides polygyrus*. Finally, they showed that scGen can use mouse data to predict perturbation responses in human cells or across other species. For such tasks, one can gain significant biological insights from the generated scRNA-seq profiles.

Our method, MichiGAN, can make the same kinds of predictions and yield the same kinds of biological insights as scGen, but we show that MichiGAN has significant benefits compared to scGen (including disentanglement and data generation performance). In addition, we show that MichiGAN can predict single-cell response to drug treatment, a biological application that was not demonstrated in the scGen paper.

## Results

### Variational autoencoders learn disentangled representations of single-cell data

Real single-cell datasets usually have unknown, unbalanced, and complex ground-truth variables, and humans cannot readily distinguish single-cell expression profiles by eye, making it difficult to assess disentanglement performance by either qualitative or quantitative evaluations. We thus first performed simulation experiments to generate balanced single-cell data with several data generating variables using the Splatter R package [63]. All the datasets were processed using the SCANPY software [68]. We measured the disentanglement performances of different methods on the simulated single-cell data using several disentanglement metrics and also provided qualitative evaluations on the learned representations using the real datasets.

**Fig. 1:**
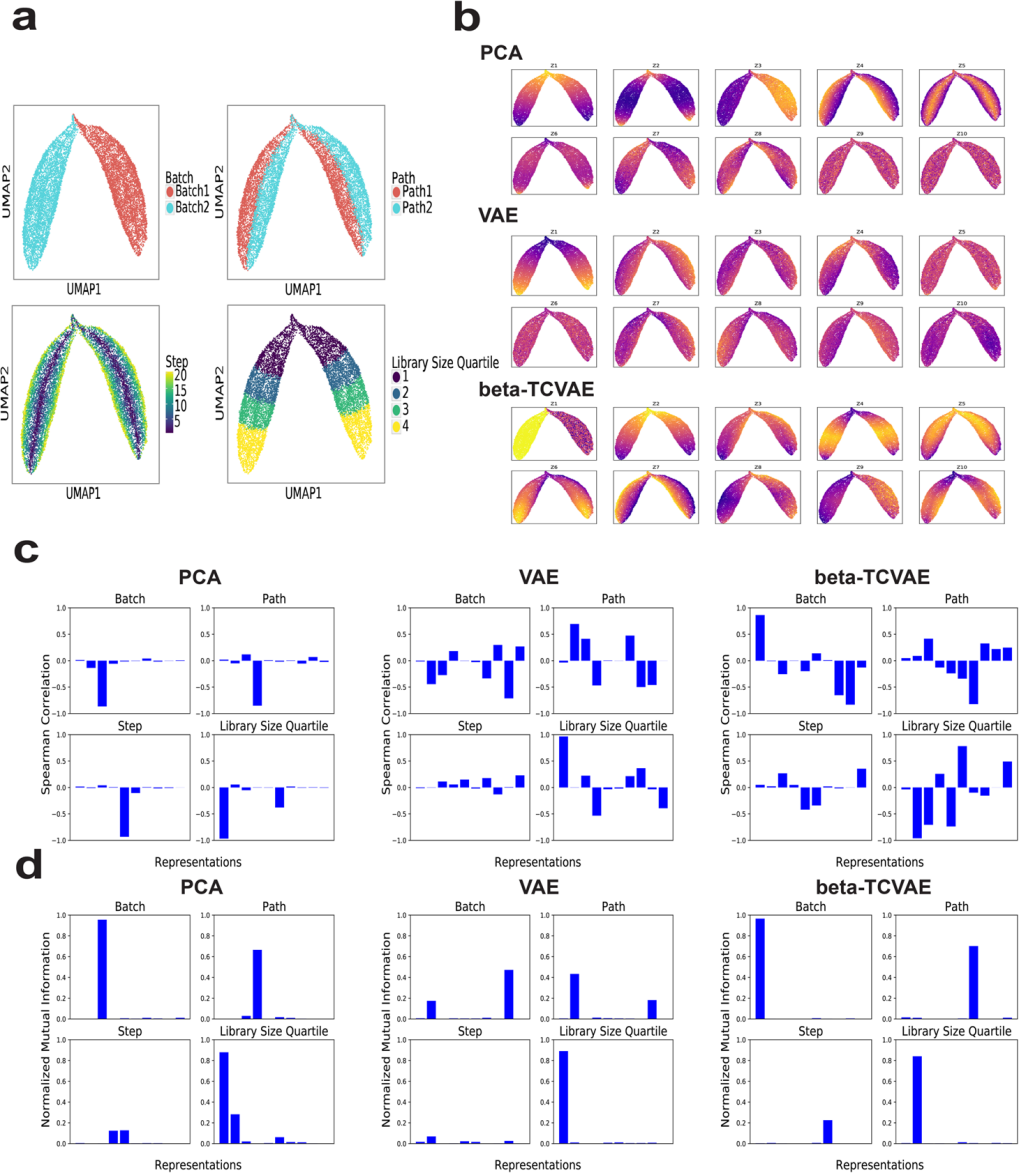
Evaluating disentanglement performance on simulated data with non-linear step. **a** UMAP plots of simulated data colored by batch, path, step, and library size quartile. **b** UMAP plots of data colored by the ten latent variables learned by PCA, VAE, and **-TCVAE. **c** Bar plots of Spearman correlations between ten latent variables and each of the four ground-truth variables for PCA, VAE, and **-TCVAE. **d** Bar plots of normalized mutual information between ten representations and each of the four ground-truth variables for PCA, VAE, and **-TCVAE

We first estimated simulation parameters to match the Tabula Muris dataset [65]. Then, we set the differential expression probability, factor location, factor scale, and common biological coefficient of variation to be (0.5,0.01,0.5,0.1). We then used Splatter [63] to simulate gene expression data of 10,000 cells with four underlying ground-truth variables: batch, path, step, and library size. Batch is a categorical variable that simulates linear differences among biological or technical replicates. Step represents the degree of progression through a simulated differentiation process, and path represents different branches of the differentiation process. We simulated two batches, two paths, and 20 steps. The batch and path variables have linear effects on the simulated expression data, while the step variable can be related either linearly or non-linearly to the simulated gene expression values. We tested the effects of this variable by separately simulating a purely linear and a non-linear differentiation process. We also included library size, the total number of expressed mRNAs per cell, as a ground truth variable. A UMAP plot of the simulated data shows that the four ground truth variables each have complementary and distinct effects on the resulting gene expression state (Fig.1a and Additional file1: Figure S1a).

We compared the disentanglement performance of three methods: probabilistic principal component analysis (PCA) [69], **-VAE, and **-TCVAE. The probabilistic PCA method assumes a linear relationship between data and representations, while VAE and **-TCVAE can learn non-linear representations. Note that we use probabilistic PCA to allow calculation of mutual information (see below). The **-TCVAE approach penalizes the total correlation of the latent representation, directly minimizing the mutual information between latent dimensions, which has been shown to significantly improve disentanglement performance on image data.

We used the three methods to learn a 10-dimensional latent representation of the simulated data (Fig.1b and Additional file1: Figure S1b). Some latent variables learned by each method showed clear relationships with the ground-truth variables. For example, the first latent variable Z1 from PCA seemed related to library size, and Z3, Z4, and Z5 were related to batch, path, and step, respectively. The VAE representations similarly showed some relationships with the ground-truth variables. Based on the UMAP plots, the latent variables from **-TCVAE appeared to show the strongest and most clear relationships with the ground-truth variables.

To quantify the disentanglement performance of the three methods, we calculated Spearman correlation and normalized mutual information between each representation and a ground-truth variable (Fig.1c, d). Spearman correlation measures the strength of monotonic relatedness between two random variables. The normalized mutual information, on the other hand, is a more general and robust metric of statistical dependence. A disentangled representation should have a bar plot with only four distinct bars in this case, indicating that each ground-truth variable was captured by exactly one latent variable. PCA showed the best performance as measured by Spearman correlation (Fig.1c), likely because the metric does not fully characterize the complex statistical dependency between true and inferred latent variables for the VAE methods, which learn more complex non-linear relationships. Based on the normalized mutual information metric, both the PCA and VAE representations achieved some degree of disentanglement, but neither approach fully disentangled all ground-truth variables. Multiple PCA representations had measurable mutual information with step and library size quartile, while multiple VAE representations identified batch and path and none of the VAE representations identified step. In contrast, exactly one **-TCVAE representation had significant mutual information for each ground-truth variable. Also, **-TCVAE was the only method with a unique representation for the non-linear step variable.

We also computed the Spearman correlation and normalized mutual information for the simulated data with linear step (Additional file1: Figure S1c-d). The results for the simulated data with linear step were similar and **-TCVAE did the best at identifying only one representation for each ground-truth variable.

We further calculated the mutual information gap (MIG) metric used in [30] and FactorVAE disentanglement metric [29] to measure disentanglement. The MIG metric is defined as the average gap between the mutual information of the two latent variables that are most related to each ground-truth variable. If there is a single latent variable that has high mutual information with each ground-truth variable, the MIG will be high. The FactorVAE metric is based on the error rate of a linear classifier that identifies which ground truth variable differs based data points using latent dimensions. In addition, we calculated a Spearman correlation gap similar to MIG. Table1 summarizes the correlation gap, FactorVAE metric, and MIG of the three models over 5 runs for the two simulated datasets. As expected from the bar charts, the PCA representations have the largest Spearman correlation gap and **-TCVAE has the largest MIG, showing the best disentanglement performance for both simulated datasets. The FactorVAE metric also shows that **-TCVAE has the best disentanglement performance. We also evaluated InfoWGAN-GP on the simulated data in Additional file1: Figure S4 and found that the representations are entangled with the ground-truth variables for simulated datasets with linear and non-linear step.

**Table 1:** Disentanglement metrics for two splatter-simulated single-cell RNA-seq datasets with four ground truth variables

		Spearman correlation gap **	FactorVAE metric **	MIG **
Linear step	PCA	**0.68** 0.00	0.35 0.01	0.54 0.00
	VAE	0.3 0.04	0.4 0.02	0.48 0.13
	**-TCVAE	0.18 0.05	**0.48** 0.03	**0.72** 0.02
Non-linear step	PCA	**0.72** 0.00	0.35 0.01	0.55 0.00
	VAE	0.27 0.07	0.41 0.02	0.43 0.08
	**-TCVAE	0.16 0.06	**0.51** 0.04	**0.66** 0.16

The mean and standard deviation over 5 runs are presented for each method. The dimensionality of the latent space was 10 for all three approaches

We also evaluated the disentanglement performance of the three methods with four latent dimensions (the same as the number of ground-truth variables), for the simulated datasets in Additional file1: Figures S8 and S9. The **-TCVAE representations still most effectively disentangle the ground-truth variables. Table2 summarizes the disentanglement metrics of the three methods with four latent dimensions. Although FactorVAE metric shows similar values for the three methods, **-TCVAE consistently has much higher MIG than PCA and VAE.

**Table 2:** Disentanglement metrics for two splatter-simulated single-cell RNA-seq datasets with four ground truth variables

		Spearman correlation gap **	FactorVAE metric **	MIG **
Linear step	PCA	0.57	0.36	0.56
	VAE	0.37	**0.44**	0.39
	**-TCVAE	**0.65**	0.33	**0.72**
Non-linear step	PCA	**0.60**	0.36	0.58
	VAE	0.4	**0.38**	0.38
	**-TCVAE	0.55	0.34	**0.73**

The dimensionality of the latent space was 4 for all three approaches

In addition, we utilized the PROSSTT package [64] to simulate three single-cell datasets. PROSSTT simulates cells undergoing a continuous process such as differentiation. As shown in Additional file1: Figures S10a, S11a and S12a, the three PROSSTT-simulated datasets have 3-, 4-, or 5-way branching trajectories, respectively. The three PROSSTT-simulated datasets also have a continuous time variable. We use three ground-truth variables (branch, time, and library size) to calculate mutual information with the learned latent variables (Additional file1: Figures S10b, S11b, and S12b). PCA and VAE have multiple latent dimensions with moderate mutual information with branch and time quartile, while **-TCVAE captures each of these quantities mostly in a single variable. We also summarized the disentanglement metrics of the three methods on the PROSSTT-simulated datasets in Table3. **-TCVAE has the highest FactorVAE metric and MIG for each of the three datasets.

**Table 3:** Disentanglement metrics for three PROSSTT-simulated single-cell RNA-seq datasets with three ground truth variables

		FactorVAE metric **	MIG **
3 trajectories	PCA	0.54	0.10
	VAE	0.58	0.08
	**-TCVAE	**0.64**	**0.27**
4 trajectories	PCA	0.59	0.12
	VAE	0.61	0.12
	**-TCVAE	**0.72**	**0.15**
5 trajectories	PCA	0.59	0.06
	VAE	0.53	0.06
	**-TCVAE	**0.62**	**0.26**

In summary, our assessment indicates that **-TCVAE most accurately disentangles the latent variables underlying single-cell data, consistent with its previously reported superior disentanglement performance on image data [30].

### GANs generate more realistic single-cell expression profiles than VAEs

We next evaluated the data generating performance of several deep generative models including VAE, **-TCVAE, and Wasserstein GAN with gradient penalty (WGAN-GP), as well as traditional methods of PCA and Gaussian mixture models (GMM) on the Tabula Muris dataset [65]. This dataset contains a comprehensive collection of single-cell gene expression profiles from nearly all mouse tissues and thus represents an appropriate dataset for evaluating data generation, analogous to the ImageNet dataset in computer vision. We also measured data generation performance on a subset of the Tabula Muris containing only cells from the mouse heart. We used two metrics to assess data generation performance: random forest error and inception score. Random forest error was introduced in the cscGAN paper [62] and quantifies how difficult it is for a random forest classifier to distinguish generated cells from real cells. A higher random forest error indicates that the generated samples are more realistic. We also computed inception score [70], a metric commonly used for quantifying generation performance on image data. Intuitively, to achieve a high inception score, a generative model must generate every class in the training dataset (analogous to recall) and every generated example must be recognizable as belonging to a particular class (analogous to precision).

**Fig. 2:**
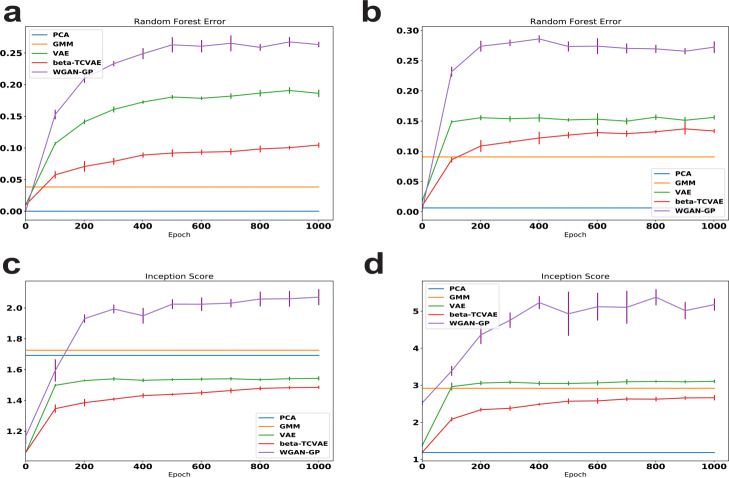
Generation performance of VAE, **-TCVAE, WGAN-GP, PCA, and GMM on the Tabula Muris heart data and the whole Tabula Muris data. **a** Random forest error for the five methods on the Tabula Muris heart data during training. **b** Random forest error for the five methods on the whole Tabula Muris data during training. **c** Inception score for the five methods on the Tabula Muris heart data during training. **d** Inception score for the five methods on the whole Tabula Muris data during training. Error bars indicate standard deviation across five runs. For clarity, the error bars for PCA and GMM are omitted because of their small and large variability

We show the random forest errors over 5 runs of VAE, **-TCVAE, and WGAN-GP during training for the Tabula Muris heart subset and the whole Tabula Muris in Fig.2a and b. We also evaluate simpler generative models, including PCA and GMM. WGAN-GP achieves the best generation performance, as measured by both metrics, on both the subset and full dataset. The deep generative models significantly outperform PCA and GMM. VAE achieves second-best generating performance and, as expected with an endeavor to pursue more disentangled representation, the quality of **-TCVAE generation is the worst of the three approaches. Figure2c, d shows the inception scores over 5 runs for the two datasets; this metric reveals the same trend as with random forest errors, indicating that WGAN-GP has the best generation performance and **-TCVAE generates the least realistic data. Additionally, the generation performance of the GAN is still significantly higher than that of the VAE even for the smaller Tabula Muris heart dataset. These results accord well with previous results from the image literature, indicating that GANs generate better samples than VAEs, and VAE modifications to encourage disentanglement come at the cost of sample quality.

### MichiGAN samples from disentangled representations without sacrificing generation performance

Having confirmed that VAEs achieve better disentanglement performance, but GANs achieve better generation performance, we sought to develop an approach that combines the strengths of both techniques. Several previous approaches have combined variational and adversarial techniques [10, 71, 72]. However, when we tested these approaches on single-cell data, we found that attempts to jointly perform variational and adversarial training compromised both training stability and generation performance. We also investigated the InfoGAN and semi-supervised InfoGAN, but found that the disentanglement performance was still significantly worse than that of the VAE approaches as shown in Additional file1: Figure S4.

We thus developed a different approach: we first train a VAE to learn a disentangled representation. Then, we use the VAE encoders latent representation *z* for each cell *x* as a given code and train a conditional GAN using the (*z*,*x*) pairs. After training, we can generate high-quality samples from the VAEs disentangled representation. Importantly, the training is no less stable than training VAE and GAN separately, and the GAN generation quality is not compromised by a regularization term encouraging disentanglement. In addition, any kind of representationfrom nonlinear methods like VAEs or linear methods like PCAcan be incorporated in our approach. Wanting to follow the convention that the names of many generative adversarial networks end with GAN, but unable to devise a compelling acronym, we named our approach MichiGAN after our institution.

**Fig. 3:**
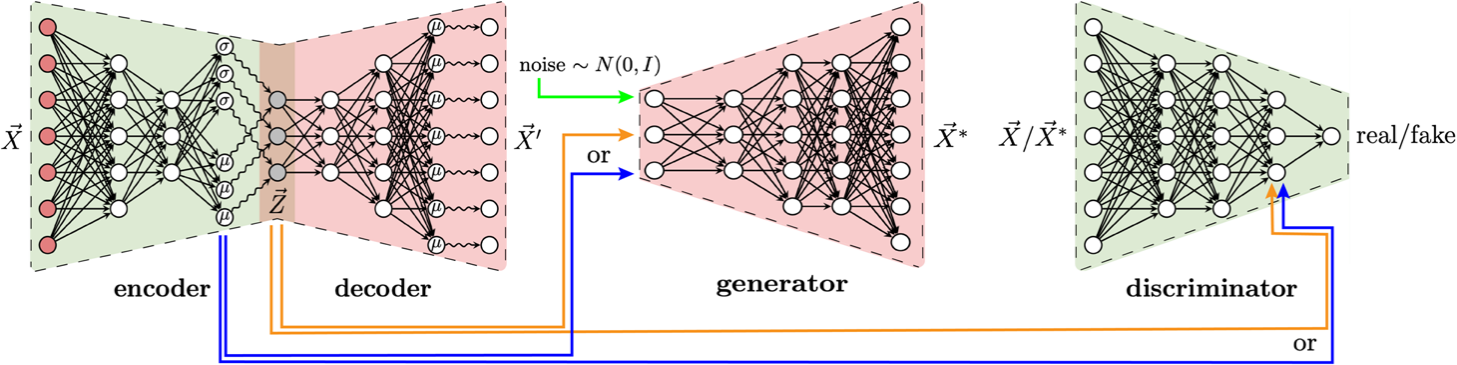
Overview of the MichiGAN architecture. We first train a model, such as **-TCVAE, to learn a disentangled representation of the real data. We then use the resulting latent codes to train a conditional GAN with projection discriminator, so that the GAN generator becomes like a more accurate decoder. Because the VAE and GAN are trained separately, training is just as stable as training each one individually, but the combined approach inherits the strengths of each individual technique. After training, we can generate high-quality samples from the disentangled representation using the GAN generator

The MichiGAN architecture is shown in Fig.3 and also summarized in Algorithm 1. We find that MichiGAN effectively achieves our goal of sampling from a disentangled representation without compromising generation quality (see results below). Several previous approaches have combined variational and adversarial techniques, including VAEGAN [10], adversarial symmetric variational autoencoder [71], and adversarial variational Bayes [72]. InfoGAN and semi-supervised InfoGAN are also conceptually related to MichiGAN, but we found that none of these previous approaches produced good results on single-cell data. While we were writing this paper, another group released a preprint with an approach called ID-GAN, which also uses a pre-trained VAE to learn a disentangled representation [40]. However, they use the reverse KL divergence framework to enforce mutual information between the VAE representation and the generated data, which we previously tested and found does work as well as a conditional GAN with projection discriminator [73]. Furthermore, ID-GAN uses a convolutional architecture and classic GAN loss for image data, whereas we use a multilayer perceptron architecture and Wasserstein loss for single-cell expression data.

Although our approach is conceptually simple, there are several underlying reasons why it performs so well, and recognizing these led us to pursue this approach. First, training a conditional GAN maximizes mutual information between the condition variable and the generated data. This is a similar intuition as the InfoGAN, but unlike InfoGAN, MichiGAN does not need to learn its own codes, and thus the discriminator can focus exclusively on enforcing the relationship between code and data. A nearly optimal discriminator is crucial for maximizing this mutual information, but the Wasserstein loss also has this requirement, and we meet it by training the discriminator 5 times for every generator update. Second, the adversarial loss allows the GAN generator to capture complex, multi-modal distributional structure that cannot be modeled by the factorized Gaussian distribution of the VAE decoder. This is particularly helpful if multiple distinct types of cells map to a similar latent code, in which case the unimodal Gaussian distribution of the VAE decoder will generate the average of these cell types. In contrast, even though the GAN generates from the same latent representation as the VAE, the GAN can fit complex, multimodal distributions by minimizing the Wasserstein distance between generated and true data distributions. Additionally, a data-dependent code (the posterior of the VAE encoder) allows the GAN to generate from a flexible latent space that reflects the data distribution, rather than an arbitrary distribution such as the commonly used standard normal. We believe this inflexibility contributes significantly to the relatively poor disentanglement performance of InfoGAN. For example, InfoGAN is highly sensitive to the number and distribution chosen for the latent codes; if classes are imbalanced in the real data but the prior has balanced classes, it cannot learn a categorical variable that reflects the true proportions.

Based on the results from our disentanglement comparison (see below), we chose to use the **-TCVAE to learn the latent representation for MichiGAN. We then use either the posterior means or the random samples from the posterior as the condition for the GANs; both choices have been utilized to evaluate disentanglement performance in previous studies [9, 29, 30].

The last step of MichiGAN involves training a conditional GAN. We found that a conditional Wasserstein GAN with projection discriminator [73] and gradient penalty [12] is most effective at enforcing the condition. We also assessed semi-supervised InfoGAN [74] and a conditional GAN based on simple concatenation, but found that these were less effective at enforcing the relationship between code and generated data (Additional file1: Figure S7) and less stable during training.

**Fig. 4:**
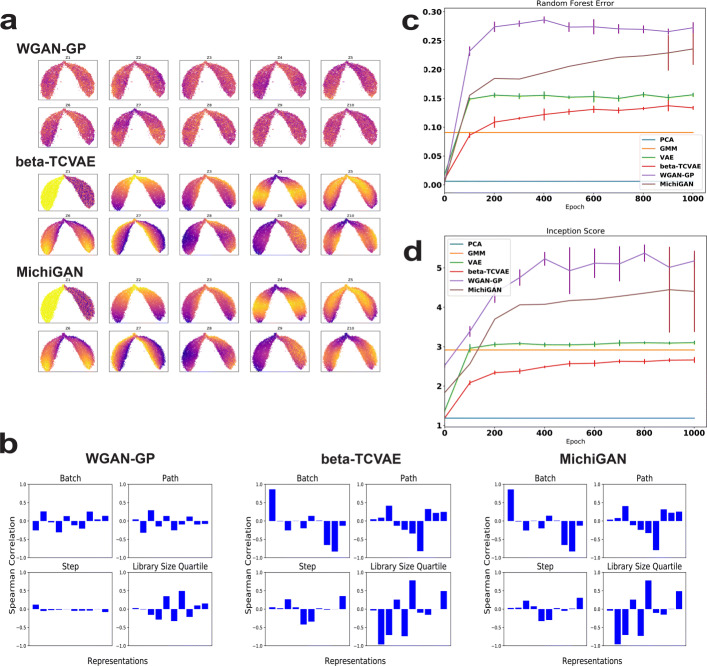
Disentanglement and generation performance of WGAN-GP, **-TCVAE, and MichiGAN. **a** UMAP plots of real data colored by the ten representations of **-TCVAE and generated data colored by the ten representations of WGAN-GP and MichiGAN on the simulated data with non-linear step. The **-TCVAE panel is reproduced from Fig.1b for clarity. **b** Bar plots of Spearman correlations between ten representations and each of the four ground-truth or inferred variables for WGAN, **-TCVAE and MichiGAN on the simulated data with non-linear step. The **-TCVAE panel is reproduced from Fig.1c for clarity. **c** Random forest error of PCA, GMM, VAE, **-TCVAE, WGAN-GP, and MichiGAN on the whole Tabula Muris data during training. **d** Inception score of PCA, GMM, VAE, **-TCVAE, WGAN-GP, and MichiGAN on the whole Tabula Muris data during training. Error bars indicate standard deviation across 5 runs. For clarity, the error bars for MichiGAN are shown only for the last 100 epochs because the convergence speed in earlier epochs is variable, and the error bars for PCA and GMM are omitted because of their small and large variability

We evaluated the MichiGAN algorithm on the simulated single-cell data with the trained **-TCVAE models. Figure4a shows the UMAP plots of real data colored by **-TCVAE latent representations and generated data colored by code using WGAN-GP and MichiGAN on the simulated data with non-linear step. The WGAN-GP representations are very entangled and none of the representations shows an identifiable coloring pattern. In contrast, the UMAP plots have consistent coloring patterns between the **-TCVAE and MichiGAN representations. Thus, the generator of MichiGAN preserves the relationship between latent code and data, effectively sampling from the disentangled representation learned by the **-TCVAE. Because there is no inference network for the generated data of either WGAN-GP or MichiGAN, we are unable to measure the mutual information for the generators. Therefore, we used Spearman correlation as an indicator of whether MichiGAN retains the relationship between disentangled latent representation and data. Figure4b also shows the bar plots of Spearman correlations between representations and variables for the three methods. We used the correlations between each representation and ground truth variables for **-TCVAE, WGAN-GP, and MichiGAN. For GAN models, we trained a *k*-nearest neighbor regressor (*k*=3) for each variable based on the real data and predicted the variables for the generated data. The WGAN-GP representations do not show large correlation with any inferred ground-truth variable. In contrast, the representations for **-TCVAE and MichiGAN show nearly identical correlations to the true variables in the real data and predicted variables in the generated data, respectively.

We also trained MichiGAN using PCA to obtain the latent code, instead of **-TCVAE. Additional file1: Figure S3a-b show the UMAP plots of real data colored by the PCA representations and generated data colored by the MichiGAN-PCA representations on the two simulated datasets. In addition, Additional file1: Figure S3c-d show nearly identical Spearman correlation bar plots between PCA and MichiGAN. MichiGAN trained with principal components preserves the relationship between the latent representations and real data, underscoring the generalizability of our approach.

We present the UMAP plots colored by the representations as well as bar plots of correlations for the simulated data with linear step in Additional file1: Figure S2a-b. The results for the simulated data with linear step also indicate that MichiGAN restores the disentanglement performance of **-TCVAE, while the WGAN-GP representations are entangled. We further summarize the correlation gaps for the three methods on two simulated datasets in Table4. For each simulated dataset, the MichiGAN and **-TCVAE have very similar correlation gaps and WGAN-GP has a very small correlation gap, as expected.

**Table 4:** Spearman correlation gap for WGAN-GP, InfoWGAN-GP, PCA, MichiGAN-PCA, VAE, **-TCVAE, and MichiGAN on the two splatter-simulated single-cell RNA-seq datasets

Model	Linear step	Non-linear step
WGAN-GP	0.07 0.02	0.10 0.06
InfoWGAN-GP	0.05 0.05	0.04 0.02
PCA	0.68 0.00	0.72 0.00
MichiGAN-PCA	0.65 0.01	0.68 0.00
VAE	0.3 0.04	0.27 0.07
**-TCVAE	0.18 0.05	0.16 0.06
MichiGAN	0.18 0.04	0.15 0.05

The mean and standard deviation are presented for each method over 5 runs

We evaluated MichiGAN on the whole Tabula Muris dataset (Fig.4c, d). MichiGAN greatly improved the data generation performance based using the disentangled representations of **-TCVAE. The random forest error of MichiGAN was larger than VAE and nearly as good as the WGAN-GP, while still generating samples from a disentangled latent space.

Additionally, we applied PCA, GMM, VAE, **-TCVAE, WGAN-GP, and MichiGAN on the pancreas endocrinogenesis dataset [66]. We obtained the cells latent time and cell cycle scores for G2M and S phases from [75]. Additional file1: Figure S13a shows the UMAP plots of data colored by latent time and the difference between G2M and S scores. The **-TCVAE method gives qualitatively more disentangled representations (Additional file1: Figure S13b), and gives much better disentanglement metrics (Additional file1: Figure S13c). In addition, Additional file1: Figure S13c also shows that MichiGAN significantly improves the data generation performance of **-TCVAE.

### MichiGAN enables semantically meaningful latent traversals

Disentangled representations of images are often evaluated qualitatively by performing latent traversals, in which a single latent variable is changed holding the others fixed. Looking at the resulting changes in the generated images to see whether only a single semantic attribute changes provides a way of visually judging the quality of disentanglement. We wanted to perform a similar assessment of MichiGAN, but single-cell gene expression values are not individually and visually interpretable in the same way that images are. We thus devised a way of using UMAP plots to visualize latent traversals on single-cell data.

We performed latent traversals using both the Tabula Muris dataset and data from the recently published sci-Plex protocol [67]. After training on the Tabula Muris dataset (Additional file1: Figure S5a), we chose a starting cell type, cardiac fibroblasts (Additional file1: Figure S5b). We then varied the value of each latent variable from low to high, keeping the values of the other variables fixed to the latent embedding of a particular cell. For the sci-Plex dataset, which contains single-cell RNA-seq data from cells of three types (A549, K562, MCF7; Additional file1: Figure S5c) treated with one of 188 drugs, we subsampled the data to include one drug treatment from each of 18 pathways by selecting the drug with the largest number of cells (Additional file1: Figure S5b). This gives one treatment for each pathway; the numbers of cells for each combination are shown in Additional file1: Table S1. We then performed latent traversals on cells with cell type MCF7 and treatment S7259 (Additional file1: Figure S5e).

**Fig. 5:**
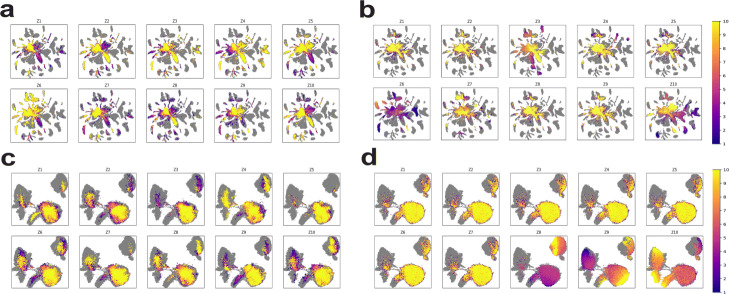
Latent traversals of WGAN-GP and MichiGAN on Tabula Muris and sci-Plex datasets. **a** UMAP plot of latent traversals of the 10 representations of latent values that generate data closest to fibroblast cells in heart within the Tabula Muris data using WGAN-GP with 128 dimensions. **b** UMAP plot of latent traversals of the 10 representations of latent values of fibroblast cells in heart within the Tabula Muris data using MichiGAN. **c** UMAP plot of latent traversals of the 10 representations of latent values that generate data closest to MCF7-S7259 cells within the sci-Plex data using WGAN-GP with 128 dimensions. **d** UMAP plot of latent traversals of the 10 representations of latent values of MCF7-S7259 cells within the sci-Plex data using MichiGAN

To visualize the traversals, we plotted each of the generated cells on a UMAP plot containing all of the real cells and colored each generated cell by the value of the latent variable used to generate it. Figure5a and b show how traversing the latent variables concentrates the generated values on each part of the UMAP plots for Tabula Muris data using the first 10 dimensions of 128-dimensional WGAN-GP and MichiGAN, respectively. Figure5c and d are the latent-traversal plots for the sci-Plex data using WGAN-GP and MichiGAN. As shown in Fig.5b, all but three of the latent variables learned by the **-TCVAE behave like noise when we traverse them starting from the fibroblast cells, a property previously noted in assessments of disentangled latent variables learned by VAEs [29]. The remaining dimensions, Z3, Z6, and Z10, show semantically meaningful latent traversals. Latent variable Z3 shows high values for mesenchymal stem cells and fibroblasts, with a gradual transition to differentiated epithelial cell types from bladder, intestine, and pancreas at lower values of Z3. This is intriguing, because the mesenchymal-epithelial transition is a key biological process in normal development, wound healing, and cell reprogramming [76]. Latent variable Z6 generates mesenchymal and endothelial cells at low values, and mammary epithelial and cardiac muscle cells at high values. Latent variable Z10 is clearly related to immune function, generating immune cells at low and medium values and traversing from hematopoietic stem and progenitor cells to monocytes, T cells, and B cells. In contrast, latent traversals in the latent space of 128-dimensional WGAN-GP (Fig.5a) do not show semantically meaningful changes along each dimension.

Figure5d also shows that MichiGANs latent traversals gives meaningful changes on the sci-Plex data. Latent variable Z8 has lower values on MCF7 cells and gradually transitions to higher values on K562 cells. In addition, latent variable Z9 also shows an A549-MCF7 transition with lower values on the A549 cells. The latent traversals of the 128-dimensional WGAN-GP, however, do not provide interpretable changes across the UMAP plot along each dimension. We also provide the latent traversals using 10-dimensional WGAN-GP for the two datasets in Additional file1: Figure S6a-b and find that the latent traversals are still not semantically meaningful.

### MichiGAN predicts single-cell gene expression changes under unseen drug treatments

One of the most exciting applications of disentangled representations is predicting high-dimensional data from unseen combinations of latent variables. We next investigated whether MichiGAN can predict single-cell gene expression response to drug treatment for unseen combinations of cell type and drug.

**Fig. 6:**
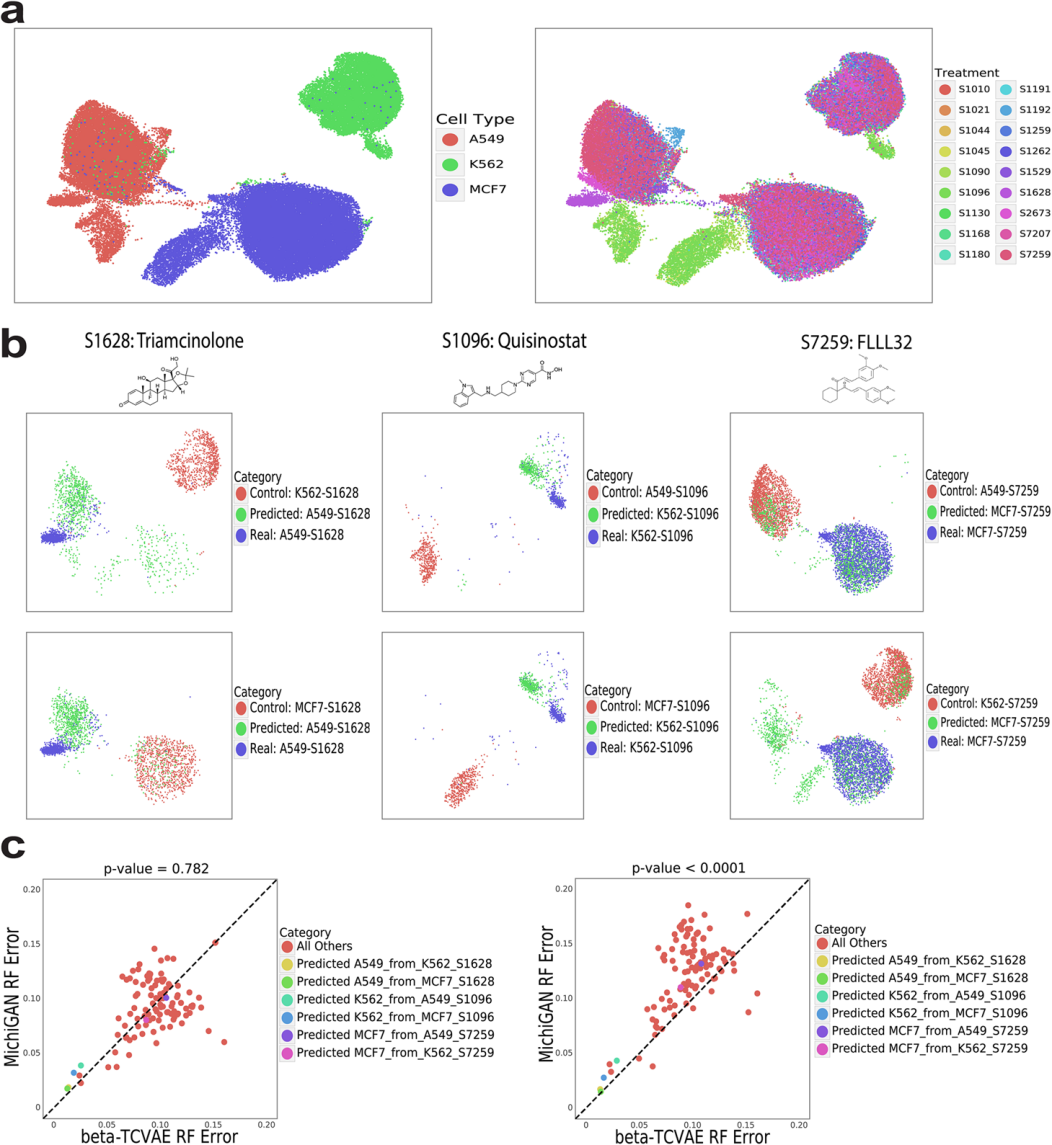
Predicting single-cell gene expression effects of unseen drugs using MichiGAN. **a** UMAP plots of sci-Plex dataset colored by cell type (left) and treatment (right). **b** UMAP plots of the predicted (green), real (blue) and control (red) cells for 6 predictions of three missing cell type/drug combinations (A549-S1628, K562-S1096, and MCF7-S7259). **c** Random forest errors between MichiGAN and **-TCVAE for all combinations. MichiGAN was trained using mean representations (left) or representations sampled from the posterior distribution (right)

We trained MichiGAN on data from the recently published sci-Plex protocol. The dataset contains single-cell RNA-seq data from cells of three types (A549, K562, MCF7), each treated with one of 188 drugs. The drug is known for each scRNA-seq profile. We subsampled the data to include one drug treatment from each of 18 pathways by selecting the drug with the largest number of cells (Fig.6a). We then have one treatment for each pathway; the numbers of cells for each combination are shown in Additional file1: Table S1. We also held out three drug/cell type combinations (A549-S1628, K562-S1096 and MCF7-S7259) to test MichiGANs out-of-sample prediction ability.

We predict single-cell gene expression for each drug/cell type combination in a two-step process. First, we estimate the mean latent difference between the target cell type and another control cell type for other treatments using either posterior means or posterior samples from the **-TCVAE encoder. We then add the average latent difference to the latent values with the same treatment and the control cell type. This latent space vector arithmetic assumes the mean cell type latent differences are homogeneous across different treatments. Note that this assumption may not hold if there is a strong interaction effect between cell type and drug treatment.

Because there are a total of three cell types, we have a total of six predictions for the three held-out drug/cell type combinations. Figure6b shows UMAP plots for these six predictions. For all six predictions, the predicted values are closer to the true drug-treated cells on the UMAP plot than the control cells used to calculate the latent vector. However, the predicted cells do not overlap with the treated cells for the combinations A549-S1628 and K562-S1096, while the two predictions for MCF7-S7259 appear to be more accurate. For both **-TCVAE and MichiGAN, we measure their random forest errors between the real and predicted cells for each combination. The random forest scatter plots for sampled representations are shown in Fig.6c. MichiGAN with sampled representations has significantly better random forest error than **-TCVAE (*p*<10^4^, one-sided Wilcoxon test) and most of the points are above the diagonal line. We also show the random forest scatter plots for mean representations in Fig.6c, which does not show significantly larger random forest errors compared to **-TCVAE (*p*>0.05, one-sided Wilcoxon test) and might be due to the remaining correlations among mean representations of **-TCVAE [19]. Thus, MichiGAN with sampled representations is able to more accurately make predictions from latent space arithmetic than **-TCVAE. However, some of the six predictions for the missing combinations show low random forest errors from both methods, and some of the predictions from MichiGAN are only marginally better than those of **-TCVAE.

### Accuracy of latent space arithmetic influences MichiGAN prediction accuracy

We next examined factors influencing the accuracy of MichiGAN predictions from latent space arithmetic. We suspected that the prediction accuracy might depend on the accuracy of the latent coordinates calculated by latent space arithmetic, which could vary depending, for example, on whether the drug exerts a consistent effect across cell types.

To investigate the reason for the difference in prediction accuracy, we developed a novel metric for assessing the accuracy of latent space arithmetic for a particular held-out cell type/perturbation combination. For a subset of the data *g*(*X*) and the latent space **(*Z*), we define the latent space entropy as:

$$ H\left\{\tau(Z), g(X)\right\} = -E_{\tau(Z)}\left[\log E_{g(X)}\left\{q_{\phi}(Z\mid X)\mid Z\right\}\right]. $$

Intuitively, *H* quantifies the concentration of *Z* with respect to *X*. We can then compare the entropy of the latent embeddings for the held-out data and the latent values predicted by latent space arithmetic by calculating *H*=*H*{**_*Fake*_(*Z*),*g*(*X*)}*H*{**_*Real*_(*Z*),*g*(*X*)}, where **_*Fake*_ is calculated by latent space arithmetic and **_*Real*_ is calculated using the encoder. The quantity *H* then gives a measure of how accurately latent space arithmetic predicts the latent values for the held-out data. If *H* is positive, then the latent space prediction is less concentrated (and thus more uncertain) than the encoding of the real data.

The quantity *H* measures how accurately latent space arithmetic predicts the latent values for the held-out data. Thus, we expect that MichiGAN should be able to more accurately predict drug/cell type combinations with a small *H*.

**Fig. 7:**
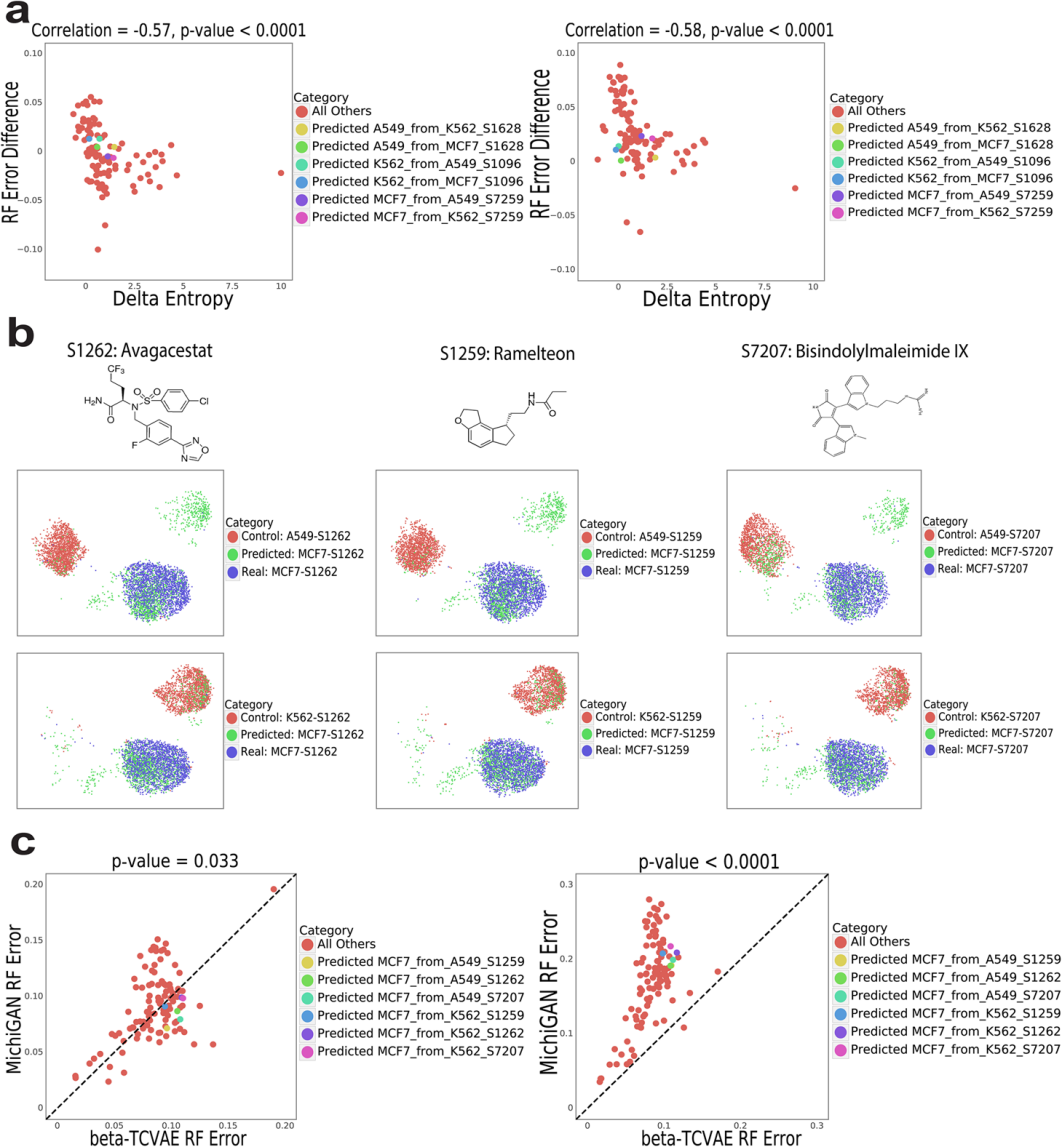
MichiGAN predicts unseen or observed combinations in the large screen sci-Plex data. **a** Scatter plots of random forest errors difference between MichiGAN and **-TCVAE versus delta entropy for MichiGAN with mean representations (left) and sampled representations (right) on the large screen sci-Plex data without three combinations of A549-S1628, K562-S1096 and MCF7-S7259. **b** UMAP plots of the predicted (green), real (blue) and control (red) cells for 6 predictions of the three missing combinations of MCF7-S1262, MCF7-S1259, and MCF7-S7207. **c** Random forest errors between MichiGAN and **-TCVAE for MichiGAN with mean representations (left) and sampled representations (right) after selecting held-out combinations with low *H*

As Fig.7a shows, *H* is significantly correlated with the difference in random forest error between MichiGAN and **-TCVAE, when sampling from either the posterior distribution of the latent representations or the posterior means. This supports our hypothesis that accuracy of the latent space arithmetic influences MichiGAN performance. To further test this, we selected the three drug/cell type combinations with the lowest overall *H* values, and re-trained the network using all combinations except these three. Figure7b shows the predicted, real and control cells for the six predictions of the three new missing combinations based on MichiGAN using sampled representations. The predicted cells (green) overlap most parts of the real cells (blue) for all six predictions. As expected, MichiGAN predicted each of these low *H* held-out combinations significantly more accurately than **-TCVAE (Fig.7c).

We also compared the performance of VAE and MichiGAN trained with VAE on the sci-Plex data after holding out the selected drug/cell type combinations with lowest overall *H* values in Additional file1: Figure S15. MichiGAN trained with VAE gives accurate prediction of the unseen combinations (Additional file1: Figure S15a), and also has significantly higher random forest error than that of VAE to predict different drug/cell type combinations using the latent space vector arithmetic algorithm (Additional file1: Figure S15b).

## Discussion

Our work provides fundamental evaluations of disentanglement performances of deep generative models on single-cell RNA-seq data. We show that combining GANs and VAEs can provide strong performance in terms of both data generation and disentanglement. MichiGAN provides an alternative to the current disentanglement learning literature, which focuses on learning disentangled representations through improved VAE-based or GAN-based methods, but rarely by combining them. Additionally, as the state of the art in disentangled representation advances, we can immediately incorporate new approaches in the MichiGAN framework, since the training of representation and GAN are completely separate.

We envision several exciting future directions. First, it would be interesting to investigate the representations learned by **-VAE or **-TCVAE across a range of biological contexts. Second, incorporating additional state-of-the-art GAN training techniques may further improve data generation quality. Additionally, there are many other biological settings in which predicting unseen combinations of latent variables may be helpful, such as cross-species analysis or disease state prediction.

## Methods

***Real scRNA-seq datasets***

The Tabula Muris dataset is a compendium of single-cell transcriptomic data from the model organism Mus musculus [65]. We processed the Tabula Muris data using SCANPY [68] and the dataset contains 41,965 cells and 4062 genes from 64 cell types. The sci-Plex dataset has three cell types treated with 188 molecules targeting 22 pathways [67]. We selected the 18 common pathways among the three cell types and chose the drug treatment from each pathway with largest number of cells. We also use SCANPY to process the data and then have 64,050 cells and 4295 genes. The pancreatic endocrinogenesis contains 3696 cells and 27,998 genes [66]. We filtered and normalized the pancreas data to 2,000 genes using the scVelo package [75]. We also and obtained the latent time and G2M and S cell cycle scores for each cell.

***Simulated scRNA-seq datasets***

To simulate data with the Splatter package, we first estimated simulation parameters to match the Tabula Muris dataset [65]. Then, we set the differential expression probability, factor location, factor scale, and common biological coefficient of variation to be (0.5,0.01,0.5,0.1). We then used Splatter [63] to simulate gene expression data of 10,000 cells with four underlying ground-truth variables: batch, path, step, and library size.

Using the PROSSTT package, we simulated 2000 genes across 10,500 (3 trajectories), 10,800 (4 trajectories), and 11,000 cells (5 trajectories). We followed the steps and parameter settings exactly as described in the PROSSTT tutorial (https://github.com/soedinglab/prosstt/blob/master/examples/many_branches_cells.ipynb), varying only the number of branches, cells, and genes.

**Variational autoencoders** VAEs have an encoder network with parameters (**), which maps the input data (*X*) to a latent space *Z*, and a decoder network parameterized by (**), which reconstructs the high-dimensional data from the latent space.

Rather than learning a deterministic function for the encoder as in a conventional autoencoder, a VAE learns the mean and variance parameters of the posterior distribution over the latent variables. However, even using a factorized Gaussian prior, the posterior is intractable. Thus, VAEs perform parameter inference using variational Bayes. Following a standard mean-field approximation, one can derive an evidence lower bound (ELBO). The objective function of VAE is to maximize the ELBO or minimize its opposite with respect to ** and **:

$$ L_{\text{VAE}} = -\text{ELBO} = E_{q(X)}\left[ - E_{q_{\phi}(Z\mid X)}p_{\theta}(X\mid Z) + \text{KL}\left\{q_{\phi}(Z\mid X)||p(Z)\right\}\right], $$

The ELBO has a nice interpretation: the first term is reconstruction error and the second term is the Kullback-Leibler divergence between the posterior and prior distributions of the latent variables (*Z*). If the prior distribution *p*(*Z*) is factorized Gaussian or uniform distribution, the KL divergence encourages the latent factors to be statistically independent, which may contribute to the good disentanglement performance of VAEs. This effect can be further enhanced by introducing a weight ** to place more emphasis on the KL divergence at the cost of reconstruction error, an approach called **-VAE [9].

****-TCVAE** The total correlation variational autoencoder (**-TCVAE) is a VAE extension that further promotes disentanglement. The KL divergence of VAE can be further decomposed into several parts:

$${} \begin{aligned} E_{q(X)}\left\{\text{KL}\left[q_{\phi}(Z\mid X)||p(Z)\right]\right\} & = \text{KL}\left[q_{\phi}(Z, X)||q_{\phi}(Z)q(X)\right] + \text{KL}\left[q_{\phi}(Z)||\prod_{j} q_{\phi}(Z_{j})\right] \\&+ \sum_{j} \text{KL}\left[q_{\phi}(Z_{j})||p(Z_{j})\right], \end{aligned} $$

The first part is referred to as the index-code mutual information (MI), the second part is the total correlation (TC), and the third part is the dimension-wise KL divergence [30]. The total correlation is the most important term for learning disentangled representations, while penalizing the two other parts does not directly improve the disentanglement performance, but increases the reconstruction error.

The **-TCVAE specifically penalizes the TC in the loss function [29, 30]:

$$L_{\beta\text{-TCVAE}} = L_{\text{VAE}} + \beta\text{KL}\left[q_{\phi}(Z)||\prod_{j} q_{\phi}(Z_{j})\right], $$

where **=0 gives the VAE loss function. There is no closed form for the total correlation of the latent representation, so **-TCVAE approximates it as follows:

$$E_{q_{\phi}(Z)}\left\{\log q_{\phi}(Z)\right\} \approx E_{q_{\phi}(Z)}\left[ \log E\left[\{ q_{\phi}(Z\mid X)\mid Z\right\}\right], $$

and

$$E_{q_{\phi}(Z_{j})}\left\{\log q_{\phi}(Z_{j})\right\} \approx E_{q_{\phi}(Z_{j})}\left[ \log E\left\{ q_{\phi}(Z_{j}\mid X)\mid Z_{j}\right\}\right]. $$

Estimating TC is difficult from a small minibatch, so we utilize the minibatch stratified sampling mentioned in [30] to estimate *E*{*q*_**_(*ZX*)*Z*} during training.

**Generative adversarial networks (GAN)** A generative adversarial network consists of a generator network *G* and a discriminator network *D*. There are many types of GANs, but we specifically focus on Wasserstein GAN with gradient penalty (WGAN-GP) [12], which significantly stabilizes GAN training. The discriminator loss function for WGAN-GP is

$$L_{\text{Discriminator}} = E_{p(Z), q(X)}\left[D(X) - D\left\{G(Z)\right\} + \lambda\left\{||\triangledown_{\widetilde{X}}D(\widetilde{X})||_{2} - 1\right\}^{2}\right], $$

where $\triangledown _{X}D$ is the gradient of the discriminator on input *X* and $\widetilde {X} = \epsilon X+ (1-\epsilon) G(Z)$ with ** sampled from a uniform distribution on [0,1]. The generator loss function for WGAN-GP is

$$L_{\text{Generator}} = E_{p(Z)}\left[D\{G(Z)\}\right]. $$

Upon convergence, WGAN-GP gives the generated data distribution *G*(*Z*) that matches the real data distribution *P*(*X*).

**InfoGAN and ssInfoGAN** The Information Maximizing Generative Adversarial Networks (InfoGAN) framework extends the regular GAN to encourage disentanglement [32]. The InfoGAN decomposes the latent variables into latent code *C* and noise *Z*. To encourage disentanglement, InfoGAN maximizes the mutual information between the latent code and the generated data. To estimate mutual information, InfoGAN relies on an additional network Q that takes generated data as input and predicts the code *Q*(*CX*) that generated the data. *Q*(*CX*) is very similar to an encoder in a VAE and estimates a posterior distribution in the same as the prior distribution of the code *p*(*C*). InfoGAN then maximizes mutual information between the code and generated data with the following loss functions for the discriminator and generator:

$$\min_{G,Q}\max_{D}L(D, G, Q) = \min_{G,Q}\max_{D}\left\{L_{\text{GAN}}(G, D) - \lambda_{\text{MI}} L_{\text{MI}}(G, Q)\right\}, $$

where *L*_MI_(*G*,*Q*)=*E*_*CP*(*C*),*XG*(*C*,*Z*)_[ log*Q*(*CX*)]+*H*(*C*) is a lower bound for the mutual information between *C* and *X* and *H*(*C*) is the entropy of the codes. We implemented InfoGAN with the Wasserstein distance, which we refer to as InfoWGAN-GP. We choose a factorized normal distribution with unit variance for *Q*(*CX*) (the unit variance stabilizes InfoGAN training [32, 36]).

InfoGAN architecture can also be extended to semi-supervised InfoGAN (ssInfoGAN), if labels are available for some or all of the data points [74]. The ssInfoGAN maximizes mutual information not only between the generated data and the codes, but also between the real data and corresponding labels. This guides the learned codes to reflect the label information.

**Conditional GAN and PCGAN** The conditional GAN extends GANs to respect the relationship between generated data and known labels [77]. There are many different network architectures for conditional GAN [77,79], but found the conditional GAN with projection discriminator (PCGAN) [73] works best. A recent paper similarly found that PCGAN worked well for single-cell RNA-seq data [62]. The original PCGAN paper mentions that the projection discriminator works most effectively when the conditional distribution *p*(*C*|*X*) is unimodal. One theoretical reason why PCGAN may be well-suited for MichiGAN is that the posterior multivariate Gaussian distributions of latent variables from VAEs are, in fact, unimodal.

In implementing the PCGAN, we do not use the conditional batch normalization or spectral normalization mentioned in [73], but instead use standard batch normalization and Wasserstein GAN with gradient penalty. Thus, we refer to this approach as PCWGAN-GP.

**MichiGAN** Algorithm 1 describes how to train MichiGAN:


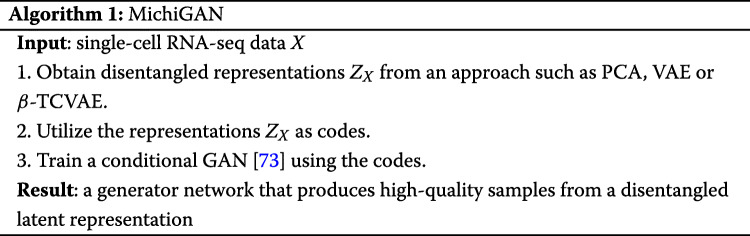


### Latent space vector arithmetic

MichiGANs ability to sample from a disentangled representation allows predicting unseen combinations of latent variables using latent space arithmetic. We perform latent space arithmetic as in [51] to predict the single-cell gene expression of unseen cell states. Specifically, suppose we have *m* cell types *C*_1_,,*C*_*m*_ and *n* perturbation *D*_1_,,*D*_*n*_. Denote *Z*(*C*_*i*_,*D*_*j*_) as the latent value corresponding to the expression data with combination (*C*_*i*_,*D*_*j*_) for 1*im* and 1*jn*. If we want to predict the unobserved expression profile for the combination $\phantom {\dot {i}\!}\left (C_{i'}, D_{j'}\right)$, we can calculate the average latent difference between cell type $\phantom {\dot {i}\!}C_{i'}$ and another cell type *C*_*k*_ in the set of observable treatments ** that $\phantom {\dot {i}\!}\Delta _{C_{i'}, C_{k}} = \int _{\Omega } \left \{Z\left (C_{i'}, D_{s}\right) - Z\left (C_{k}, D_{s}\right)\right \}dP(s)$ and then use the latent space $\phantom {\dot {i}\!}Z\left (C_{k}, D_{j'}\right)$ of observed combination $\phantom {\dot {i}\!}\left (C_{k}, D_{j'}\right)$ to predict

$$ \widehat{Z}\left(C_{i'}, D_{j'}\right) = Z\left(C_{k}, D_{j'}\right) + \Delta_{C_{i'}, C_{k}}. $$

The predicted $\phantom {\dot {i}\!} \widehat {Z}\left (C_{i'}, D_{j'}\right)$ is further used to generate predicted data of the unseen combination. The predicted latent space assumes the average latent difference across observed treatments is equal to the latent difference of the unobserved treatment, which may not hold if there is a strong cell type effect for the perturbation.

### Disentanglement metrics

#### Mutual information

Following [30], we measure the disentanglement performance of the representations using mutual information gap (MIG). Denote *p*(*V*_*k*_) and *p*(*XV*_*k*_) as the probability of a ground-truth variable *V*_*k*_ and the conditional probability of the data *X* under *V*_*k*_. Given $q_{\phi }\left (Z_{j}, V_{k}\right) = \int _{X} p\left (V_{k}\right)p\left (X\mid V_{k}\right)q_{\phi }\left (Z_{j}\mid X\right)dx$, the mutual information between a latent variable *Z*_*j*_ and a ground-truth variable *V*_*k*_ is defined as

$$I\left(Z_{j}, V_{k}\right) = E_{q_{\phi}\left(Z_{j}, V_{k}\right)}\left\{\log \int_{X\in \mathcal{X}_{V_{k}}}q_{\phi}\left(Z_{j}\mid X\right)p\left(X\mid V_{k}\right)dX\right\} + H\left(Z_{j}\right), $$

where $\mathcal {X}_{V_{k}}$ is the support of *p*(*XV*_*k*_) and *H*(*Z*_*j*_) is the entropy of *Z*_*j*_. Due to the different variabilities of the ground-truth variables, the normalized mutual information is better to be used with a normalization term of *H*(*V*_*k*_), the entropy of *V*_*k*_. The posterior distribution *q*_**_(*Z*_*j*_*X*) is obtained from the encoder (for VAEs) or the derived posterior distribution for probabilistic PCA [80]. With *K* ground truth variables {*V*_1_,,*V*_*k*_}, the mutual information gap (MIG) is further defined as

$$\text{MIG} = \frac{1}{K}\sum_{k=1}^{K}\frac{1}{H(V_{k})}\left\{I\left(Z_{j^{(k)} }, V_{k}\right) - \max_{j\neq j^{(k)}}I(Z_{j}, V_{k})\right\}, $$

where *j*^(*k*)^= arg max*jI*(*Z*_*j*_,*V*_*k*_).

The MIG metric is the average difference between largest and the second largest normalized mutual information value across all ground-truth variables. Intuitively, this indicates how much each ground truth variable is captured by a single latent variable. As described in [30], the MIG metric has the axis-alignment property and is unbiased for all hyperparameter settings.

#### FactorVAE metric

For completeness, we also calculated the disentanglement metric introduced in the FactorVAE paper [29]. In each of multiple repetitions, we first randomly choose a ground-truth variable and then generate data, keeping this variable fixed and other variables at random. We normalize each dimension by the empirical standard deviation over the whole data and choose the dimension with the lowest empirical variance. The dimension with the lowest empirical variance and the fixed ground-truth variable are then used as (*x*,*y*) pairs to train a majority vote classifier. The FactorVAE disentanglement metric is defined as the accuracy of the resulting classifier.

#### Spearman correlation

Inspired by the MIG metric, we also utilized Spearman correlation to quantify disentanglement performance. Although Spearman correlation is a more restricted metric of statistical dependence than mutual information, it has the advantage that it can be computed without a distributional estimate of a latent representation, which is not available for GAN models. Given the Spearman correlation *S*=cor(*Z*_*j*_,*V*_*k*_) between inferred representation *Z*_*j*_ and ground truth variable *V*_*k*_, we define the corresponding correlation gap as $|\text {cor}\left (Z_{j^{(k)} }, V_{k}\right)| - \max _{j\neq j^{(k)}}|\text {cor}\left (Z_{j}, V_{k}\right)|$, where *j*^(*k*)^= arg max*j*|cor(*Z*_*j*_,*V*_*k*_)|.

### Generation metrics

#### Random forest error

We follow the random forest error metric introduced in the cscGAN paper [62] to quantify how difficult it is for a random forest classifier to distinguish generated cells from real cells. A higher random forest error indicates that the generated samples are more realistic. We randomly sample 3000 cells and generate 3000 additional cells. Then we train a random forest classifier on the 50 principal components of the 6000 cells to predict each cell to be a real or fake cell. We train with 5-fold cross validation and report the average error across the 5 folds.

#### Inception score

We also define an inception score metric similar to the one widely used in evaluating performance on image data [70]. Intuitively, to achieve a high inception score, a generative model must generate every class in the training dataset (analogous to recall) and every generated example must be recognizable as belonging to a particular class (analogous to precision). We train a random forest classifier on 3000 randomly sampled real cells to predict their cell types. Based on the trained cell-type classifier, we are able to predict the probabilities of being different cell types for each generated cell. We then input the predicted probabilities to the calculations of the inception score.

### Tuning ** values in **-TCVAE

The ** value is a hyperparameter in the **-TCVAE model that controls the relative importance of penalizing the total correlation of the learned representation. Because ** is a hyperparameter in an unsupervised learning approach (no ground truth is available in general), there is no direct way to pick a single best value for **. This is not a problem unique to the **-TCVAE, but is a general challenge with any unsupervised learning approach. Our best recommendation is to choose a value in the range of 1050 and use whatever biological prior knowledge is available, such as annotations of cell time point, condition, or cell type, to qualitatively assess the disentanglement of representations for different values. One of the best things one can hope for with unsupervised learning algorithms is that the results are robust to different hyperparameter settings. To show that this is true in this case, we measured disentanglement performance of VAE and **-TCVAE for **=10 and 50 on the simulated datasets as shown in Additional file1: Figure S14. We found that **-TCVAE with **=10 or 50 consistently gives a higher mutual information gap (MIG) than VAE. In short, even if you do not choose the perfect value of **, it is still better to use **-TCVAE than VAE.

### Implementation

The VAE-based methods use multilayer perceptron (MLP) units and have two fully connected (FC) hidden layers with 512 and 256 neurons, followed by separate parameters for mean and variance of the latent representation. The first two hidden layers in the decoder have 256 and 512 neurons, while the last layer gives mean gene expression and has the same number of neurons as the number of genes. Each hidden layer utilizes batch normalization, activated by Rectified Linear Unit (ReLU) or Leaky ReLU. Each hidden layer employs dropout regularization, with a dropout probability of 0.2. We also experimented with three hidden layers for the VAE encoders, but found that the training became unstable. This is consistent with a previous report [60] that found most VAEs for biological data have only two hidden layers. The GAN-based methods also use MLP for both generator and discriminator. There are three FC hidden layers with 256, 512, and 1024 neurons as well as three hidden layers with 1024, 512, and 10 neurons from data to output. The hidden layers of GANs also have Batch Normalization and ReLU or Leaky ReLU activation. The generator uses dropout regularization with dropout probability of 0.2 for each hidden layer. The VAE-based methods are trained with Adam optimization, while the GAN-based methods are trained with Adam and the gradient prediction method [81]. All the hyperparameters of each method on different datasets are tuned for the optimal results.

We trained all models for 1000 epochs and used 10 latent variables. We used **=10 for **-TCVAE on all of the splatter-simulated single-cell RNA-seq datasets, except that we used **=5 for **-TCVAE with 4 latent dimensions on the simulated data with linear step. We used **=50 for **-TCVAE on the PROSSTT-simulated datasets and pancreas dataset. For the two real scRNA-seq datasets, we used **=100. We used 118-dimensional Gaussian noise for MichiGAN. All models were implemented in TensorFlow.

## Supplementary Information

**Additional file 1** Tables S1 and Figures S1S15

**Additional file 2** Review history

## Data Availability

MichiGAN code is available at a DOI-assigning repository Zenodo (10.5281/zenodo.4728278) [82] and at GitHub (https://github.com/welch-lab/MichiGAN) [83] under GNU General Public License v3.0. Detailed documentation and a Jupyter notebook demonstrating how to use the package are available on the GitHub page. The tabula muris data [65] supporting the conclusions of this article are available in the tabula-muris Python GitHub repository, https://github.com/czbiohub/tabula-muris. The large sci-Plex data [67] are available on GEO (GSM4150378, https://www.ncbi.nlm.nih.gov/geo/query/acc.cgi?acc=GSM4150378). The pancreas endocrinogenesis dataset [66] as well as its cells latent time and cell cycle scores for G2M and S phases are available from the examples of the scVelo package [75] at https://scvelo.readthedocs.io/Pancreas.html. The R package splatter is implemented on R version 3.6.1. The PROSSTT package is available at https://github.com/soedinglab/prosstt. The deep generative models and performance metrics are based on TensorFlow version 1.14.0 and Python 3.6. Declarations
