# Supplementary material for: MichiGAN: sampling from disentangled representations of single-cell data using generative adversarial networks
Source: Genome Biol. 2021 May 20;22:158. doi: 10.1186/s13059-021-02373-4 (PMC8139054; doi:10.1186/s13059-021-02373-4)
Supplement: Supplementary file 1 — Additional file 1 Tables S1 and Figures S1S15 [file 13059_2021_2373_MOESM1_ESM.pdf]

RESEARCH

Supplementary material for "MichiGAN: sampling from disentangled representations of single-cell data using generative adversarial networks"

Hengshi Yu and Joshua D. Welch*

*Correspondence:
welchjd@umich.edu
Full list of author information is available at the end of the article

Table S1: Number of cells for each the cell type/drug combinations selected from the sci-Plex dataset

		Cell Type		
Pathway	Treatment	A549	K562	MCF7
Protein Tyrosine Kinase	S1010	1014	800	1548
Angiogenesis	S1021	791	506	1626
PI3K/Akt/mTOR	S1044	700	800	1530
Others	S1045	882	787	1849
Cytoskeletal Signaling	S1090	1934	643	1928
Epigenetics	S1096	879	450	1688
Apoptosis	S1130	216	694	79
Neuronal Signaling	S1168	1009	1006	1984
Stem Cells & Wnt	S1180	934	934	1854
Endocrinology & Hormones	S1191	957	982	1945
DNA Damage	S1192	808	803	1392
GPCR & G Protein	S1259	1061	1083	1682
Proteases	S1261	874	964	1759
Cell Cycle	S1529	1499	739	1077
Metabolism	S1628	902	1162	1899
MAPK	S2673	724	730	1823
TGF-beta/Smad	S7207	2195	759	1431
JAK/STAT	S7259	2846	875	2014

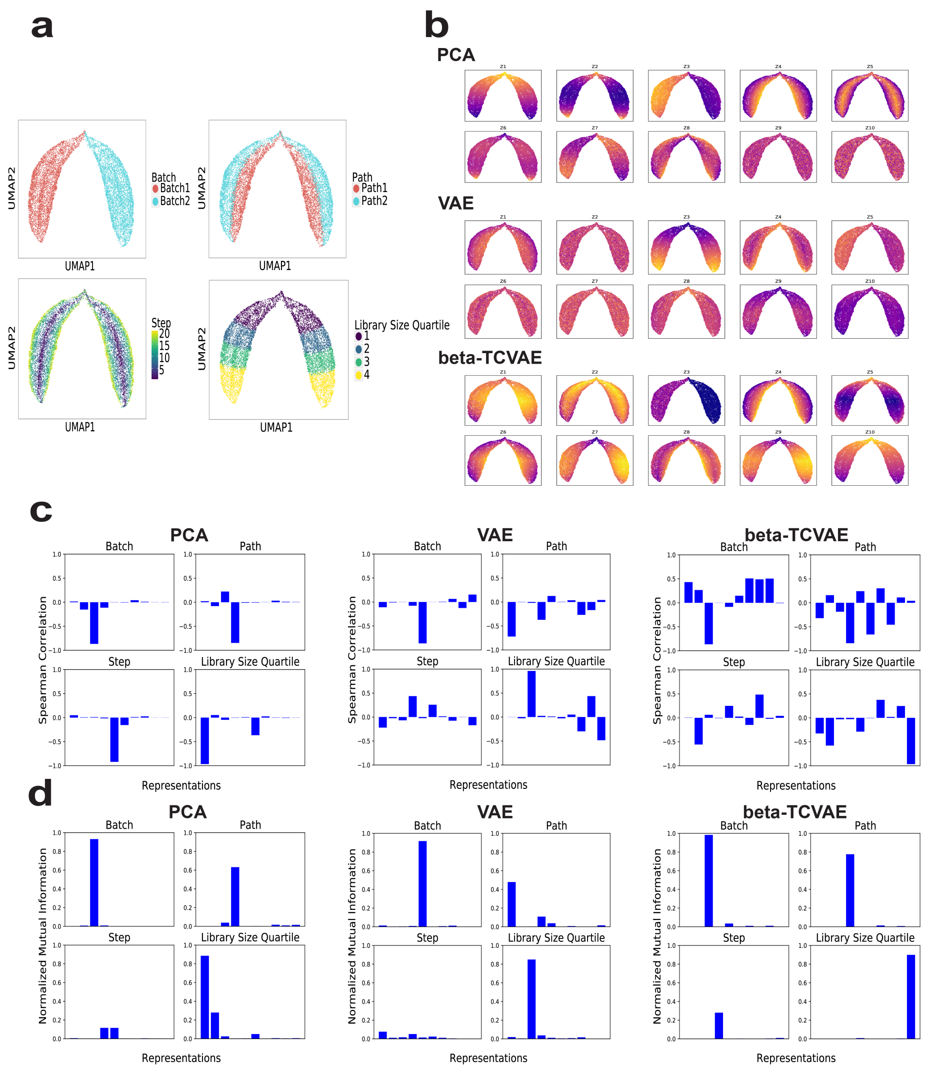

Fig. S1 Evaluating disentanglement performance on simulated dataset with linear step. **a** UMAP plots of simulated data colored by batch, path, step and library size quartile. **b** UMAP plots of data colored by the ten latent variables learned by PCA, VAE and β-TCVAE. **c** Bar plots of Spearman correlations between ten latent variables and each of the four ground-truth variables for PCA, VAE and β-TCVAE. **d** Bar plots of normalized mutual information between ten latent variables and each of the four ground-truth variables for PCA, VAE and β-TCVAE.

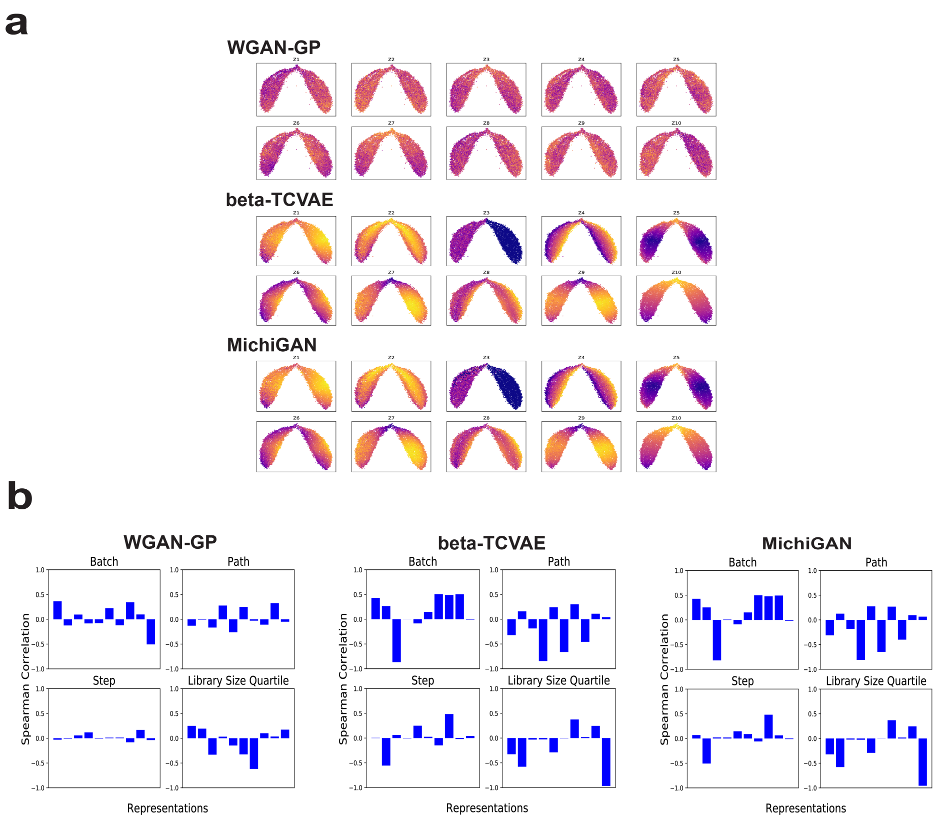

Fig. S2 Disentanglement and generation performance of WGAN-GP, β-TCVAE and MichiGAN. **a** UMAP plots of real data colored by the ten representations of β-TCVAE and generated data colored by the ten representations of WGAN-GP and MichiGAN on the simulated data with linear step. The β-TCVAE panel is reproduced from Fig. S1b for clarity. **b** Bar plots of Spearman correlations between ten representations and each of the four ground-truth or inferred variables for WGAN, β-TCVAE and MichiGAN on the simulated data with linear step. The β-TCVAE panel is reproduced from Fig. S1c for clarity.

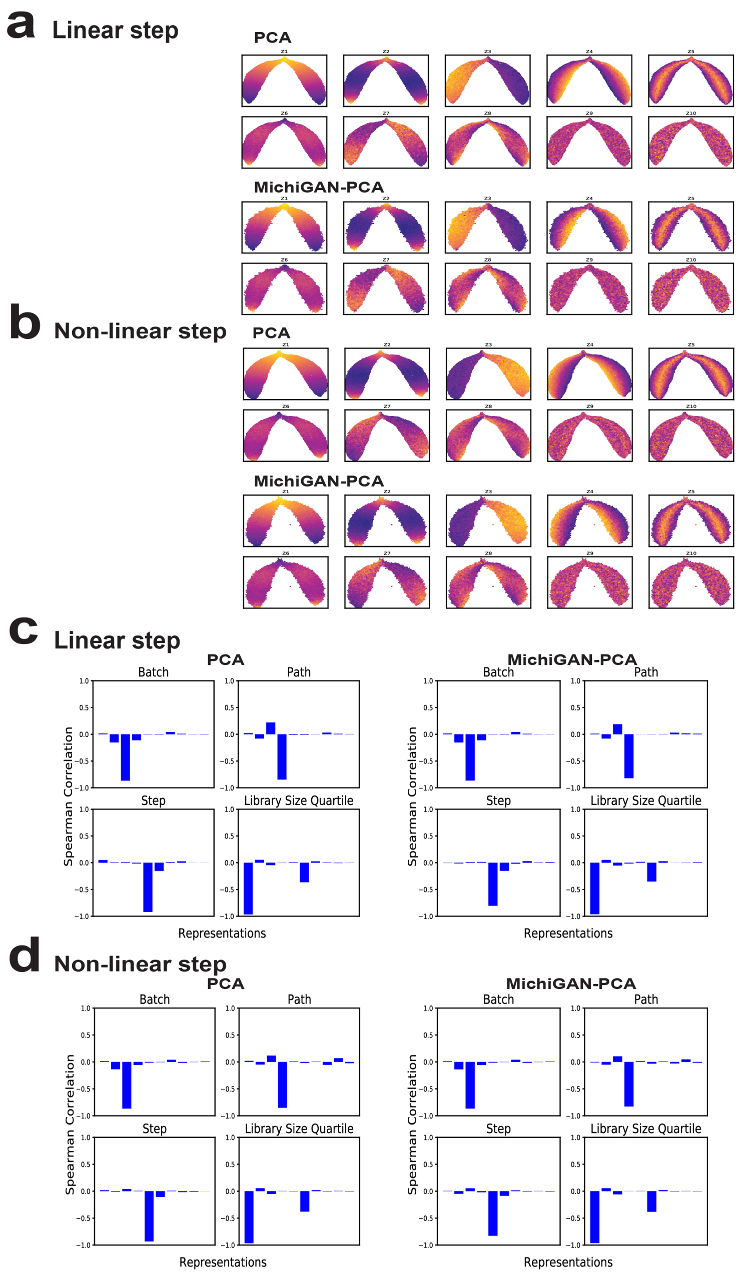

Fig. S3 Disentanglement performance of PCA and MichiGAN-PCA. **a** UMAP plots of real data colored by ten representations of PCA and generated data colored by the MichiGAN-PCA representations on the simulated data with linear step. **b** UMAP plots of real data colored by ten representations of PCA and generated data colored by the MichiGAN-PCA representations on the simulated data with non-linear step. **c** Bar plots of Spearman correlations between ten representations and each of the four ground-truth or inferred variables for PCA and MichiGAN-PCA on the simulated data with linear step. **d** Bar plots of Spearman correlations between ten representations and each of the four ground-truth or inferred variables for PCA and MichiGAN-PCA on the simulated data with non-linear step. The PCA panels are reproduced from Fig. 1b-c and Fig. S1b-c for clarity.

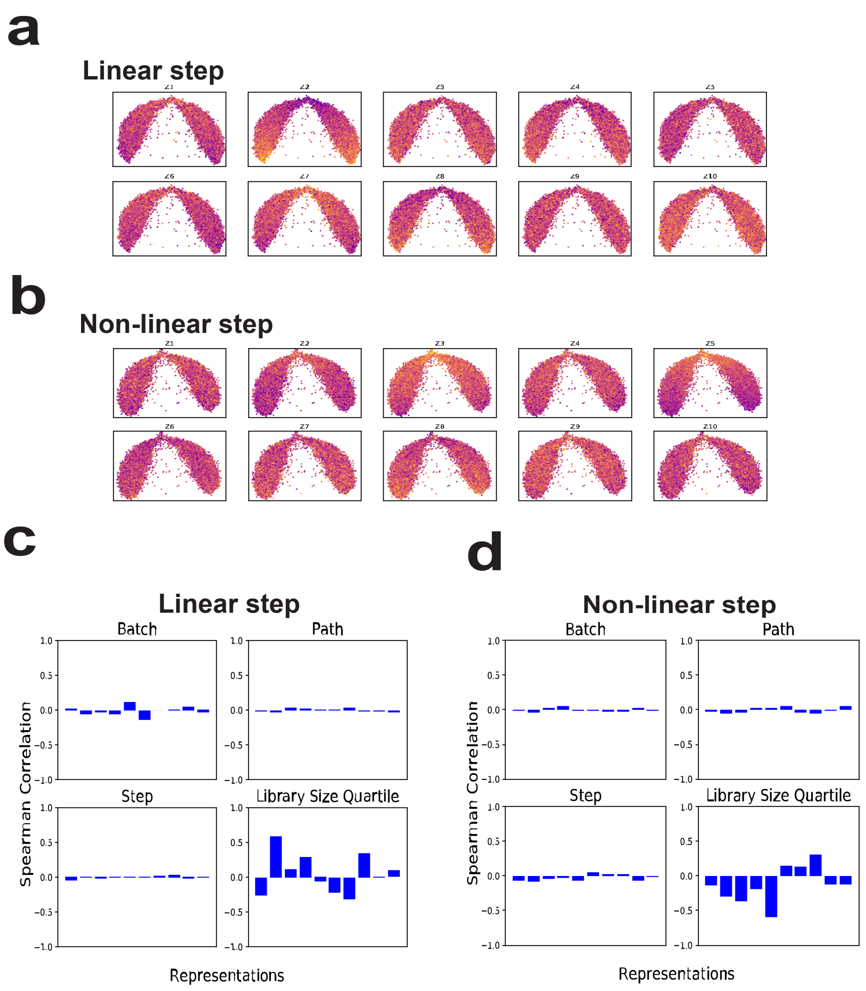

Fig. S4 Representations learned by InfoWGAN-GP from the simulated single-cell data. **a** UMAP plots of the simulated data with linear step colored by the ten representations learned by InfoWGAN-GP. **b** UMAP plots of the simulated data with non-linear step colored by the ten representations learned by InfoWGAN-GP. **c** Bar plots of Spearman correlations between ten representations and each of the four ground-truth variables for InfoWGAN-GP on the simulated data with linear step. **d** Bar plots of Spearman correlations between ten representations and each of the four ground-truth variables for InfoWGAN-GP on the simulated data with non-linear step.

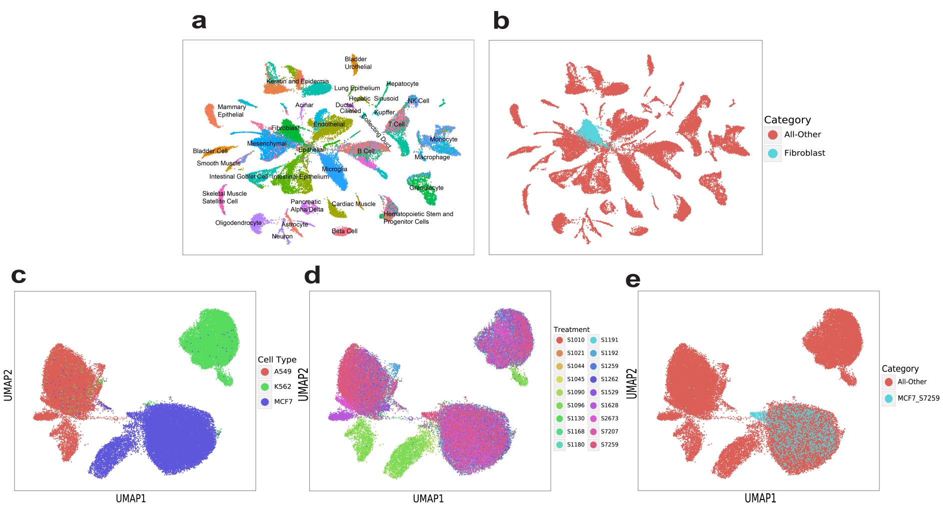

Fig. S5 The whole Tabula Muris data and the large sci-Plex data. **a** UMAP plot of the whole Tabula Muris data colored by cell type. **b** UMAP plot of the 2026 fibroblast cells in heart within the whole Tabula Muris data. **c** UMAP plot of the sci-Plex data colored by cell type. **d** UMAP plot of the sci-Plex data colored by drug treatment. **e** UMAP plot of the 2014 cells with MCF7 cell type and S7259 treatment within the sci-Plex data. For clarity, **c** and **d** are reproduced from Fig. 6a.

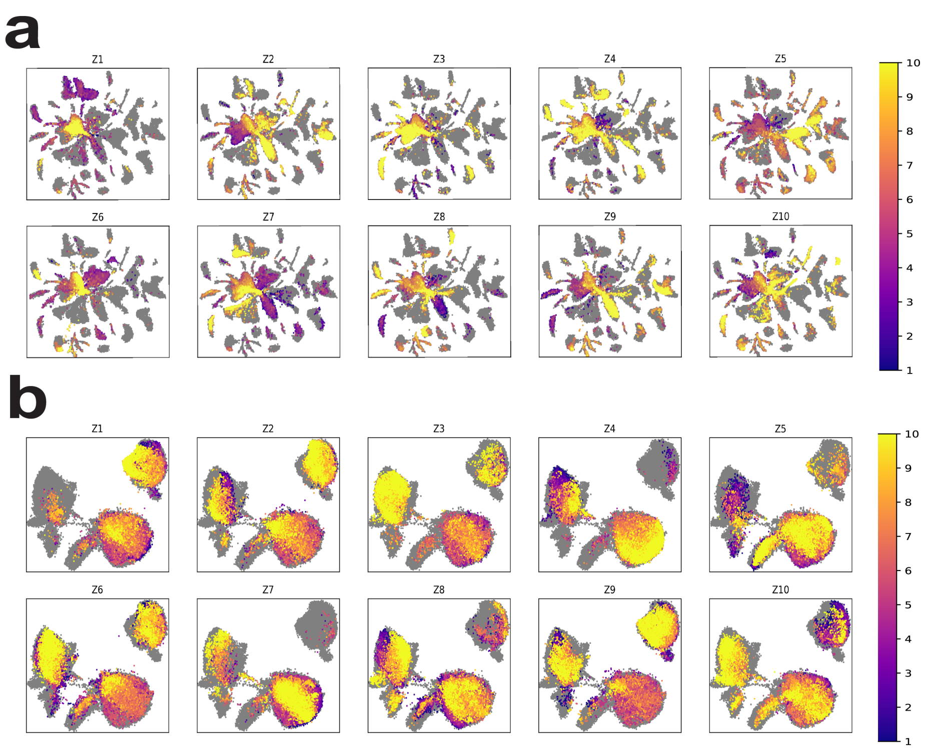

Fig. S6 UMAP plots of data generated via latent traversals. **a** UMAP plot of latent traversals of the 10 representations of latent values that generate data closest to fibroblast cells in heart within the Tabula Muris data using WGAN-GP with 10 dimensions. **b** UMAP plot of latent traversals of the 10 representations of latent values that generate data closest to MCF7-S7259 cells within the sci-Plex data using WGAN-GP with 10 dimensions.

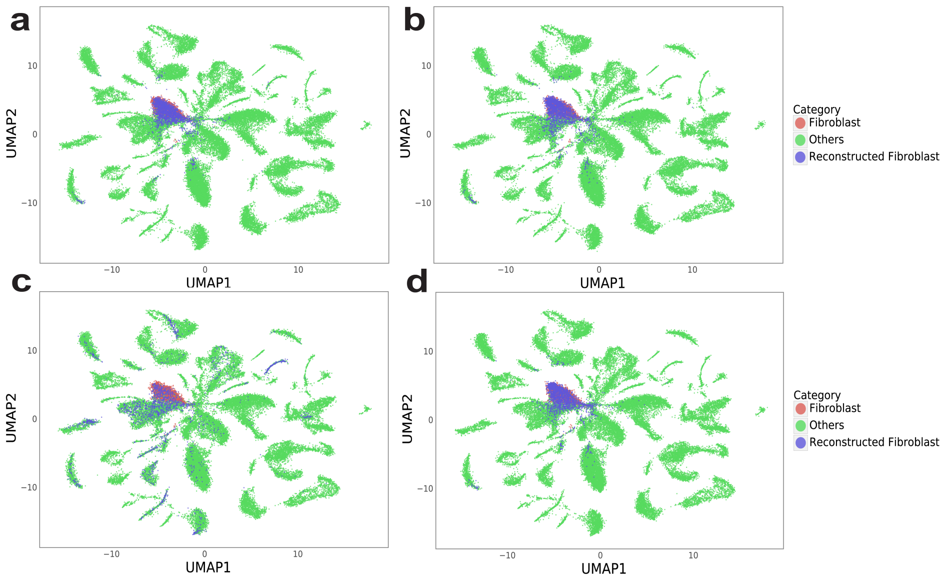

Fig. S7 Comparison of conditional GAN strategies. **a** UMAP plots of reconstructed cardiac fibroblast cells using β-TCVAE. **b** UMAP plots of reconstructed cardiac fibroblast cells using MichiGAN with PCWGAN-GP. **c** UMAP plots of reconstructed cardiac fibroblast cells using MichiGAN with ssInfoWGAN-GP. **d** UMAP plots of reconstructed cardiac fibroblast cells using MichiGAN with CWGAN-GP.

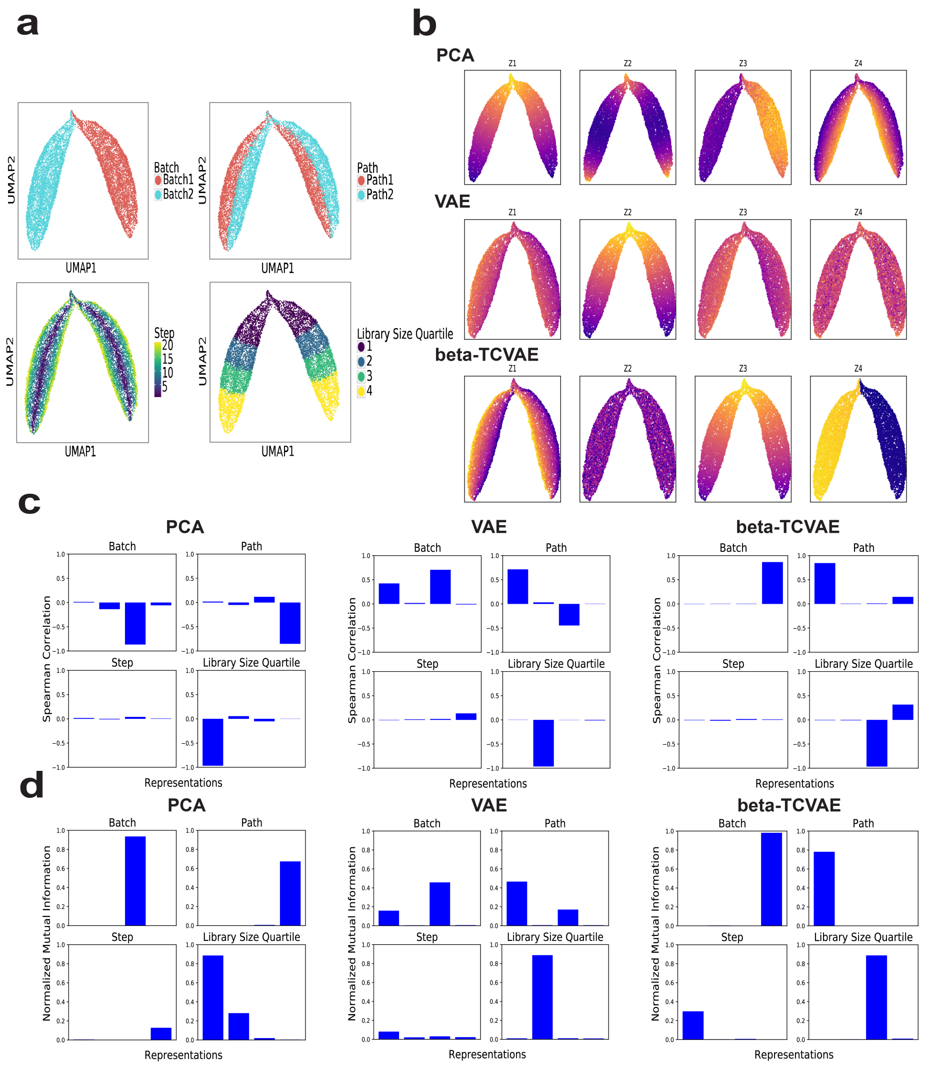

Fig. S8 Evaluating disentanglement performance on simulated dataset with non-linear step. **a** UMAP plots of simulated data colored by batch, path, step and library size quartile. **b** UMAP plots of data colored by the four latent variables learned by PCA, VAE and β-TCVAE. **c** Bar plots of Spearman correlations between four latent variables and each of the four ground-truth variables for PCA, VAE and β-TCVAE. **d** Bar plots of normalized mutual information between four latent variables and each of the four ground-truth variables for PCA, VAE and β-TCVAE. For clarity, **a** is reproduced from Fig. 1a.

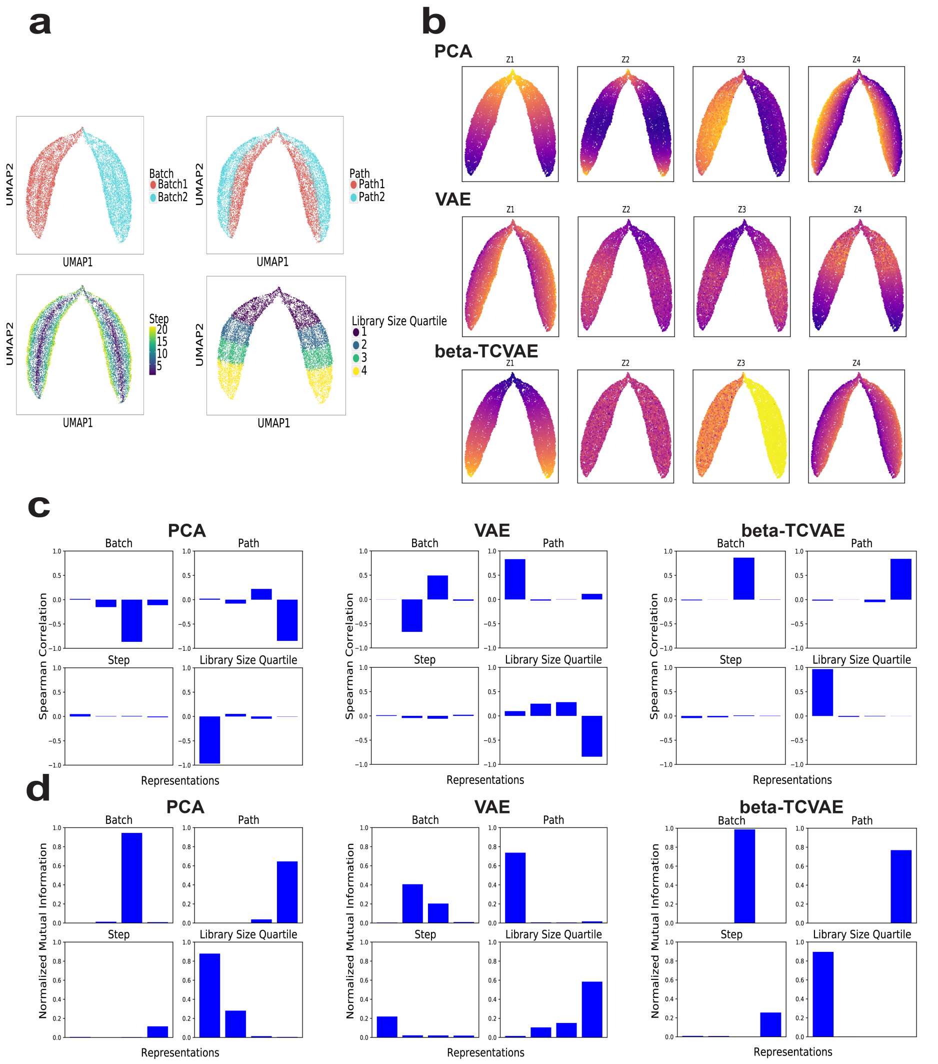

Fig. S9 Evaluating disentanglement performance on simulated dataset with linear step. **a** UMAP plots of simulated data colored by batch, path, step and library size quartile. **b** UMAP plots of data colored by the four latent variables learned by PCA, VAE and β-TCVAE. **c** Bar plots of Spearman correlations between four latent variables and each of the four ground-truth variables for PCA, VAE and β-TCVAE. **d** Bar plots of normalized mutual information between four latent variables and each of the four ground-truth variables for PCA, VAE and β-TCVAE. For clarity, **a** is reproduced from Fig. S1a.

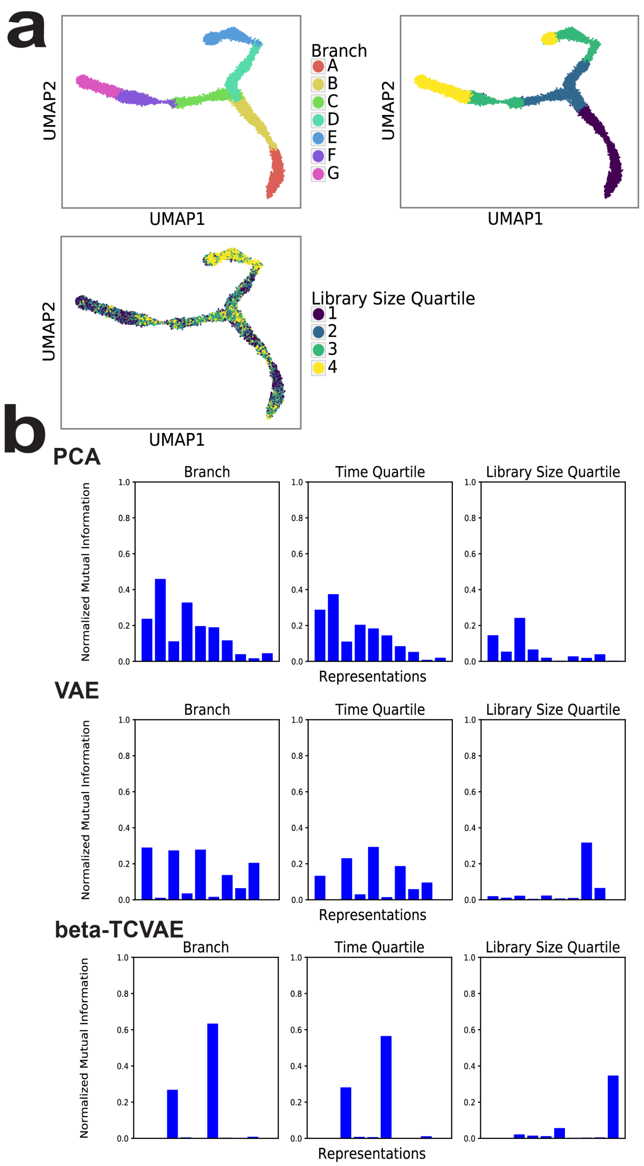

Fig. S10 Evaluating disentanglement performance on simulated dataset by PROSSTT with 3 main trajectories. **a** UMAP plots of simulated data colored by branch, time quartile and library size quartile. **b** Bar plots of normalized mutual information between ten latent variables and each of the three ground-truth variables for PCA, VAE and β-TCVAE.

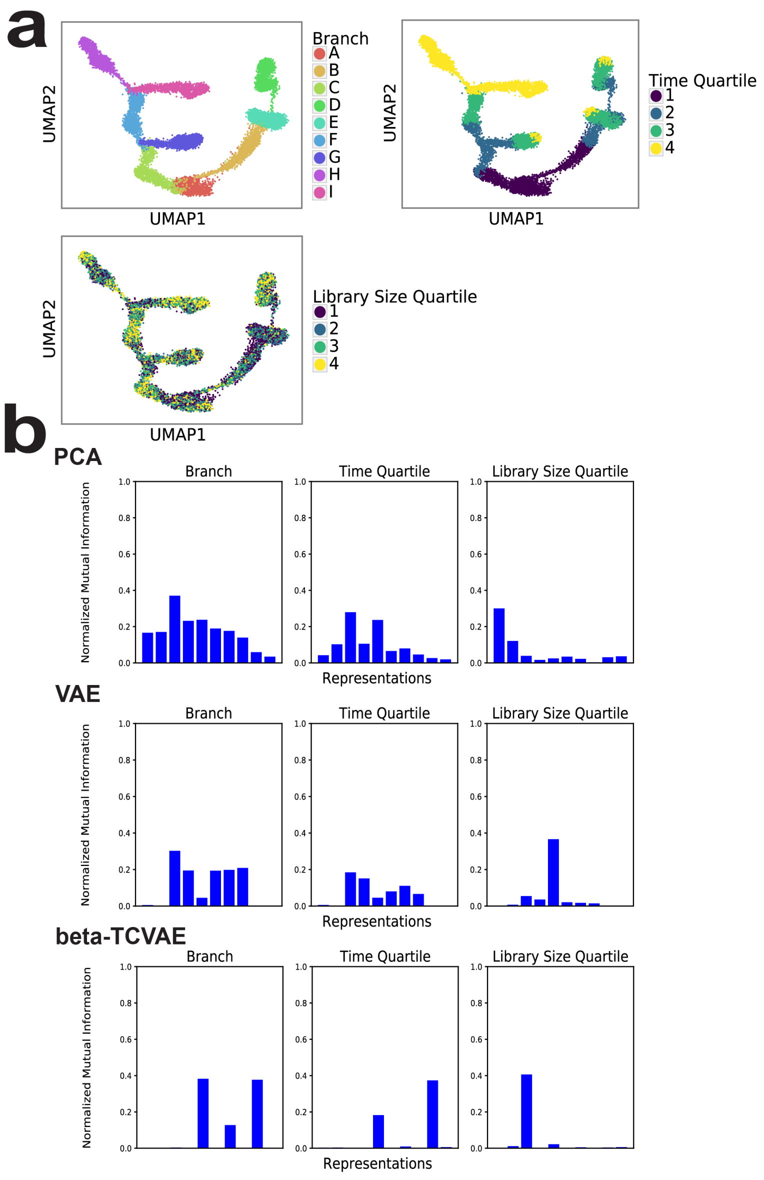

Fig. S11 Evaluating disentanglement performance on simulated dataset by PROSSTT with 4 main trajectories. **a** UMAP plots of simulated data colored by branch, time quartile and library size quartile. **b** Bar plots of normalized mutual information between ten latent variables and each of the three ground-truth variables for PCA, VAE and β-TCVAE.

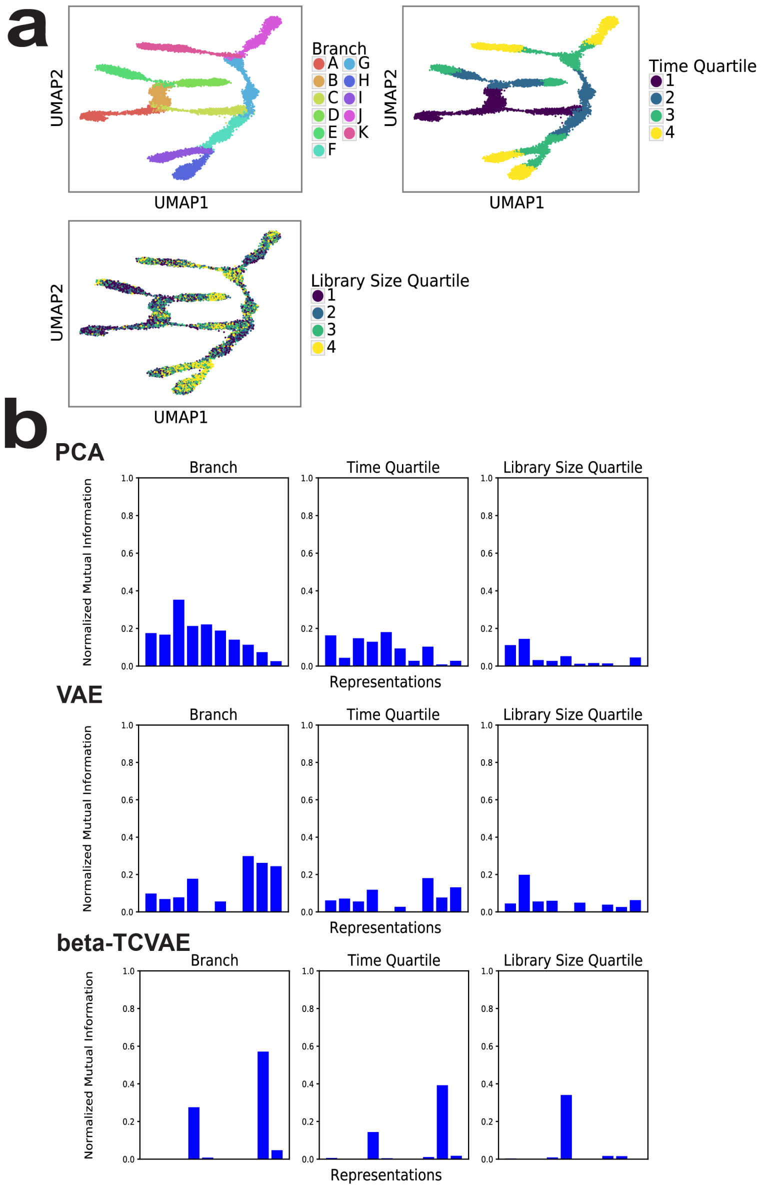

Fig. S12 Evaluating disentanglement performance on simulated dataset by PROSSTT with 5 main trajectories. **a** UMAP plots of simulated data colored by branch, time quartile and library size quartile. **b** Bar plots of normalized mutual information between ten latent variables and each of the three ground-truth variables for PCA, VAE and β-TCVAE.

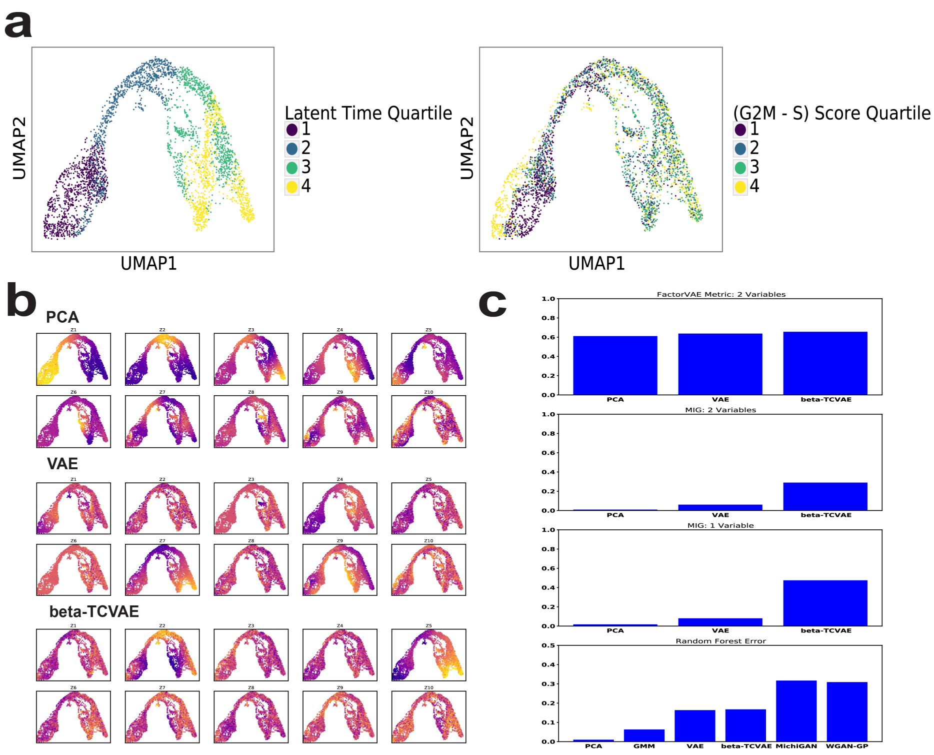

Fig. S13 Disentanglement and generation performance on pancreas endocrinogenesis. **a** UMAP plots of data colored by latent time quartile and the quartile of the difference between the G2M and S cycle scores. **b** UMAP plots of data colored by the ten latent variables learned by PCA, VAE and β-TCVAE. **c** Bar plots of FactorVAE metric, MIG for PCA, VAE and β-TCVAE, as well as random forest error for PCA, GMM, VAE, β-TCVAE, MichiGAN and WGAN-GP. For clarity, the 2 variables refer to latent time quartile and (G2M - S) score quartile, and the 1 variable means only latent time quartile.

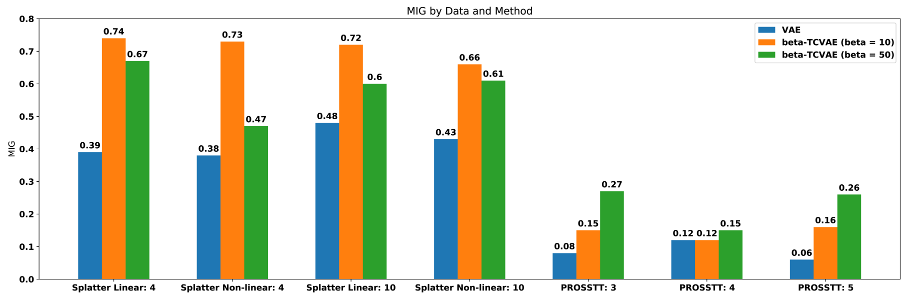

Fig. S14 Robustness of disentanglement performance: MIG of VAE and β-TCVAE ($\beta = 10, 50$) on simulated datasets.

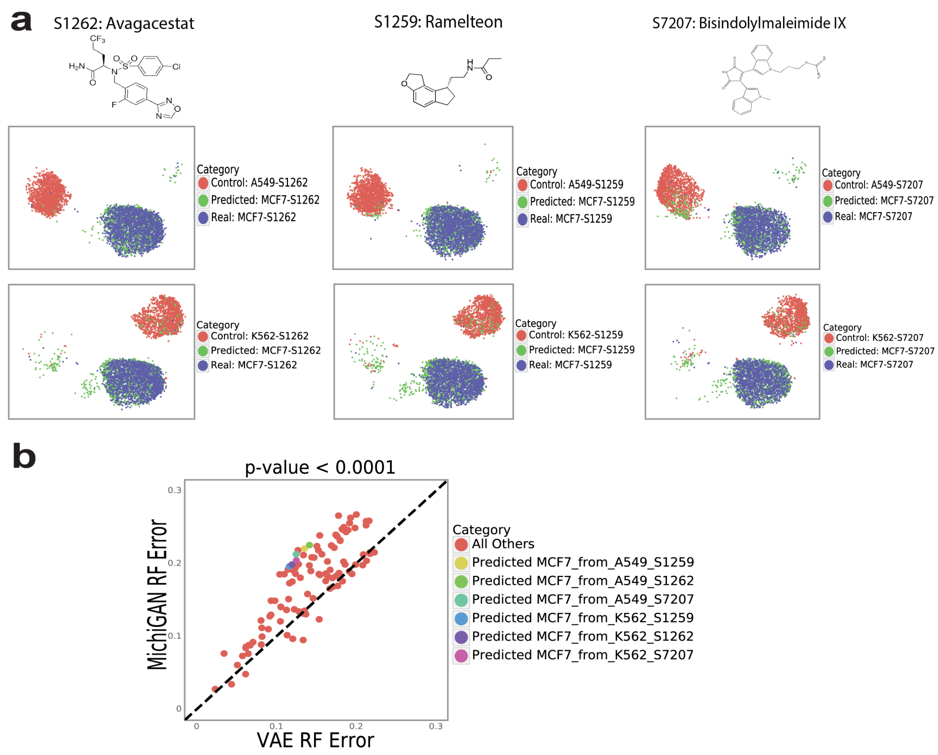

Fig. S15 MichiGAN based on VAE predicts unseen or observed combinations in the sci-Plex dataset. **a** UMAP plots of the predicted (green), real (blue) and control (red) cells for 6 predictions of the three missing combinations of MCF7-S1262, MCF7-S1259 and MCF7-S7207. **b** Random forest errors values for MichiGAN trained on VAE and VAE alone after selecting held-out combinations with low ΔH.
