# Supplementary material for: MichiGAN: sampling from disentangled representations of single-cell data using generative adversarial networks
Source: Genome Biol. 2021 May 20;22:158. doi: 10.1186/s13059-021-02373-4 (PMC8139054; doi:10.1186/s13059-021-02373-4)
Supplement: Supplementary file 2 — Additional file 2 Review history [file 13059_2021_2373_MOESM2_ESM.docx]

Review History

**First round of review**

**Reviewer 1**

**Comments to author:**

This study explores the potential of leveraging some recent machine-learning algorithms (VAE, GAN) on single-cell RNA-seq data analysis, focusing on deriving a set of deconvoluted, semantically meaningful latent variables and on generating "realistic" scRNA-seq data from latent variables. Aspects on machine-learning were well addressed and described.

Major comments:

*	Biological motivation/application is not clear. What biological contexts can this approach be applied to? If to generate out-of-sample data, how meaningful (accurate) the results really are? How much real knowledge can one learn from the scRNA-seq data "imputed" by MichiGAN? If to infer meaningful latent variables, can a protocol be developed to determine which specific latent variables are semantically meaningful (and hence worth performing latent transversal)?

*	The disentanglement power of beta-TCVAE elucidated by this study is interesting. This work did a good job at demonstrating the potential of applying beta-TCVAE to scRNA-seq data analysis. However, beta-TCVAE itself is not originated from this work.

Minor comments:

*	The power of the GAN (WGAN-GP specifically) itself is not as clearly elucidated, even though the intention to capture the non-linear mapping makes sense.

*	Fig. 1d, although beta-TCVAE disentanglement results look cleaner, path and step were both mapped to the same latent variable z7? Can this be explained?

*	Page 6 bottom, "This suggests that the adversarial training process may be able to better utilize smaller datasets." Is this already known in the ML literature? If so, citations should be included.

*	Page 7, "We thus developed a different approach: we first train a VAE to learn a dis-entangled representation." Has this hybrid VAE+GAN approach already been attempted in image analysis/synthesis? If so, citations need to be included.

*	Page 8, "We also assessed semi-supervised InfoGAN [72] and a conditional GAN based on simple concatenation, but found that these were much less effective at enforcing the relationship between code and generated data." For completeness, shall the result be included in the supplemental materials?

*	Fig. 4c, MichiGAN seems to converge slower than other approaches. Why is that?

In summary, this work presented some interesting exploration of how cutting-edge VAE and GAN approaches can potentially help advance scRNA-seq data analysis. The descriptions on ML principles, experiments, metrics and comparisons with various approaches are informative and interesting. However, the work did not clearly define a biological domain/problem, where the computational work can clearly impact.

**Reviewer 2**

**Comments to author:**

The authors first assessed the disentanglement and the generation performance of GANs and VAEs on a simulated scRNA-seq dataset and proposed MichiGAN to combines the strengths of VAEs and GANs. They applied MichiGAN to simulated and two real scRNA-seq datasets, and showed the disentangled representations can capture the aspects of cellular identity and predict unseen combinations of cell states. Overall, I feel this method somewhat interesting, but still have quite a few critical concerns, which need to be fully addressed.

Major:

1. Why were exactly 10 latent dimensions adopted? What are the results for 4 latent dimensions, for there are only 4 ground-truth factors in the simulated data?

2. Fig.1b clearly shows the disentangled property of features learned by PCA, the linear relationship is very straightforward through eyeballing. And the Spearman Correlation displayed in Fig.1c confirms this. The disentangled property seems better persevered by PCA than beat-TCVAE in linear measurement. The authors may use the PCA representation as the input of GAN as a baseline.

3. The conclusions about GANs and VAEs were made by comparing their performances on only one simulated dataset, which is not sufficient for validation. And only two real data were tested.

4. Besides, the observations and rules on the simulated data may not hold when it comes to real scRNA-seq datasets. And how do other methods (like WGAN and beta-VAE) perform on the real datasets you used for the case-study?

5. How do the other traditional methods other than GANs or VAEs perform in your cases? Is it necessary to apply a quite complicated deep generative neural network to scRNA-seq datasets, which usually have only a limited number of very tight cell clusters? What is the point that other simpler methods could not achieve?

6. The authors show the Random Forest Error of VAE, beta-TCBAE, WGAN-GP, MichiGAN in Fig.4. How robustness of this result? The robustness of MichiGAN should be explored since it is a two-stage method.

7. Moreover, since the authors use different β values for different experiments, how to select this hyperparameter in real applications?

8. The authors demonstrated single-cell gene expression could predict unseen drug or drug combinations by MichiGAN. It seems this could be done in a simple way. Also the effectiveness should be compared with other related ones (e.g., The RF Error of WGAN-GP).

Minor:

9. The authors may consider to put some technical details in the method description to make the result section easy to follow.

**Authors Response**

**Point-by-point responses to the reviewers’ comments:**

*We thank the reviewers for their constructive comments and suggestions, which have significantly improved the clarity and rigor of our manuscript. We have included point-by-point responses to your comments below.*

Reviewer #1: This study explores the potential of leveraging some recent machine-learning algorithms (VAE, GAN) on single-cell RNA-seq data analysis, focusing on deriving a set of deconvoluted, semantically meaningful latent variables and on generating "realistic" scRNA-seq data from latent variables. Aspects on machine-learning were well addressed and described.

Major comments:

* Biological motivation/application is not clear. What biological contexts can this approach be applied to? If to generate out-of-sample data, how meaningful (accurate) the results really are? How much real knowledge can one learn from the scRNA-seq data "imputed" by MichiGAN? If to infer meaningful latent variables, can a protocol be developed to determine which specific latent variables are semantically meaningful (and hence worth performing latent transversal)?

*At a high level, our paper addresses the problems of (1) identifying the factors that underlie biological differences among cells and (2) predicting how cellular transcriptomic profiles vary along these underlying latent variables. These are very general methodological challenges, analogous to cell clustering or cell trajectory inference--both of which can be applied to a wide range of biological questions.*

*Our work builds upon a recent Nature Methods paper (Lotfollahi et al., 2019) that utilized a simple VAE (which they called scGen) to predict single-cell perturbation responses. Lotfollahi et al. showed several specific biological contexts in which this type of approach is useful. First, they predicted the cell-type-specific gene expression changes induced by treating immune cells with lipopolysaccharide. Second, they predicted the cell-type-specific changes that occur when intestinal epithelial cells are infected by Salmonella or Heligmosomoides polygyrus. Finally, Lotfollahi et al. showed that scGen can use mouse data to predict perturbation responses in human cells or across other species. For such tasks, one can gain significant biological insights from the generated scRNA-seq profiles. We have added a clearer description of this motivation and previous work to the revised manuscript.*

*Our method, MichiGAN, makes the same kinds of predictions and yields the same kinds of biological insights as scGen, but we show that MichiGAN has significant benefits compared to scGen (including disentanglement and data generation performance). In addition, we show that MichiGAN can predict single-cell response to drug treatment, a biological application that was not demonstrated in the scGen paper. In the revised manuscript, we also analyzed an additional real dataset showing that disentangled representations capture developmental stage and cell cycle stage when applied to single-cell data from the developing pancreas. We have updated the text to clarify these points.*

*Both quantitative metrics and latent traversals can help identify the semantic meanings of latent variables. Metrics such as Spearman correlation and mutual information, which we used in the paper, can indicate latent variables related to cell metadata, such as time point, annotated cell type, or biological condition (e.g., perturbed vs. control). Latent traversal is also commonly used to determine the semantic meanings of latent variables from models trained on image data. We performed such latent traversals on our single-cell data. For example, our latent traversal analysis of the Tabula Muris data revealed latent variables related to differences among immune and epithelial cell types.*

* The disentanglement power of beta-TCVAE elucidated by this study is interesting. This work did a good job at demonstrating the potential of applying beta-TCVAE to scRNA-seq data analysis. However, beta-TCVAE itself is not originated from this work.

*The reviewer is correct that we did not create beta-TCVAE ourselves. Instead, the innovation in our paper is (1) showing that beta-TCVAE can improve disentanglement performance on single-cell data, not just the image data for which it was originally designed; and (2) combining the disentangled representations learned by beta-TCVAE with a generative adversarial network to improve data generation performance. We previously did cite the beta-TCVAE paper in the text, but have updated the text to further clarify the nature of our contribution and its distinction from previous work.*

Minor comments:

* The power of the GAN (WGAN-GP specifically) itself is not as clearly elucidated, even though the intention to capture the non-linear mapping makes sense.

*The power of GAN approaches is simply their ability to achieve superior data generation performance. Our experiments showed that GANs, including WGAN-GP and MichiGAN, achieved much better data generation performance than VAE-based methods, as measured by both random forest error and inception score. We note that, although the reasons for this difference in performance are not fully understood, previous papers have suggested that the adversarial loss used in GAN models better captures multimodal distributional structure, whereas the reconstruction error term in the VAE objective function blends together distinct distributional modes (which is visible as blurring in reconstructed image data). We have clarified these points in the text. The end of the paragraph 1 now reads:*

*“GANs and VAEs possess complementary strengths and weaknesses: GANs generate much better samples than VAEs [11], but VAE training is much more stable and learns more useful ``disentangled'' latent representations [12]. GANs outperform VAEs in generating sharp image samples [10], while VAEs tend to generate blurry images [13]. GAN training is generally less stable than VAE training, but some recent derivations of GAN like Wasserstein GAN [14, 15, 16] significantly improve the stability of GAN training, which is particularly helpful for non-image data.”*

* Fig. 1d, although beta-TCVAE disentanglement results look cleaner, path and step were both mapped to the same latent variable z7? Can this be explained?

*A common definition of disentanglement is that there is only one latent variable related to each factor of variation. This definition does not require that the mapping from underlying factors of variation to latent variables must be one-to-one. In the case of our simulated data, for either path or step variables, the latent variable Z7 is the only variable with quantifiable mutual information, thus satisfying this definition of disentanglement.*

* Page 6 bottom, "This suggests that the adversarial training process may be able to better utilize smaller datasets." Is this already known in the ML literature? If so, citations should be included.

*After re-training the VAE and GAN models 5 times each on the smaller dataset, this observation did not replicate. GANs still significantly outperform VAEs on both the large and small datasets, but the gap between GAN and VAE is not larger for the smaller dataset. Thus, we have modified the previous statement to “The generation performance of the GAN is still significantly higher than that of the VAE even for the smaller Tabula Muris heart dataset”.*

* Page 7, "We thus developed a different approach: we first train a VAE to learn a dis-entangled representation." Has this hybrid VAE+GAN approach already been attempted in image analysis/synthesis? If so, citations need to be included.

*Although other papers have attempted to combine VAEs and GANs in some ways, our approach is unique. We previously summarized this prior work at the end of the methods section. For clarity, we have moved the description of prior work to the results section, before where we introduce the MichiGAN framework. The paragraph reads:*

*“The MichiGAN architecture is shown in Fig. 3 and also summarized in Algorithm 1. We find that MichiGAN effectively achieves our goal of sampling from a disentangled representation without compromising generation quality (see results below). Several previous approaches have combined variational and adversarial techniques, including VAEGAN [13], adversarial symmetric variational autoencoder [71], and adversarial variational Bayes [72]. InfoGAN and semi-supervised InfoGAN are also conceptually related to MichiGAN, but we found that none of these previous approaches produced good results on single-cell data. While we were writing this paper, another group released a preprint with an approach called ID-GAN, which also uses a pre-trained VAE to learn a disentangled representation [43]. However, they use the reverse KL divergence framework to enforce mutual information between the VAE representation and the generated data, which we previously tested and found does work as well as a conditional GAN with projection discriminator [73]. Furthermore, ID-GAN uses a convolutional architecture and classic GAN loss for image data, whereas we use a multilayer perceptron architecture and Wasserstein loss for single-cell expression data.”*

* Page 8, "We also assessed semi-supervised InfoGAN [72] and a conditional GAN based on simple concatenation, but found that these were much less effective at enforcing the relationship between code and generated data." For completeness, shall the result be included in the supplemental materials?

*To demonstrate the advantages of PCGAN for enforcing the relationship between code and generated data, we used different methods to generate cardiac fibroblast cells (Fig. S7). This figure shows that, when conditioning on the latent codes from fibroblast cells, MichiGAN with PCWGAN-GP most effectively preserves the relationship between code and data. In addition, we found that PCWGAN-GP training is significantly more stable than that of semi-supervised InfoGAN and conditional GAN based on simple concatenation. In our experiments, the ssInfoWGAN-GP and CWGAN-GP failed to converge (due to vanishing/exploding gradients) in four out of five training runs, while PCWGAN-GP converged successfully in all five runs we performed. We have mentioned this point in the text.*

* Fig. 4c, MichiGAN seems to converge slower than other approaches. Why is that?

*After re-running the MichiGAN 5 times, following the suggestion of Reviewer 2, the convergence of inception score metrics looks more similar to that of the WGAN-GP (see Fig. 4c-d). The random forest error still appears to converge slightly more slowly than the WGAN-GP. While it is not entirely clear what accounts for this difference, the added conditional information incorporated into the GAN loss may change the training landscape. Unlike WGAN-GP, MichiGAN must not only generate data points that look realistic, but must also ensure that the generated points match their codes. This more complex task could cause the training to require more iterations before the data generation reaches its maximum value. However, the key point is that MichiGAN eventually achieves a significantly higher generation performance than the VAE approaches.*

In summary, this work presented some interesting exploration of how cutting-edge VAE and GAN approaches can potentially help advance scRNA-seq data analysis. The descriptions on ML principles, experiments, metrics and comparisons with various approaches are informative and interesting. However, the work did not clearly define a biological domain/problem, where the computational work can clearly impact.

*As discussed in our response to your first comment, our paper addresses the problems of (1) identifying the factors that underlie biological differences among cells and (2) predicting how cellular transcriptomic profiles vary along these underlying latent variables. These are very general methodological challenges, analogous to cell clustering or cell trajectory inference--both of which can be applied to a wide range of biological questions.*

*Our work builds on the scGen paper recently published in Nature Methods, which utilized a simple VAE to predict single-cell perturbation responses. MichiGAN can perform the same types of biological tasks as scGen: predicting out-of-sample perturbation response shared among cell types, cell-type-specific response or response across species. In addition, we also showed that MichiGAN can predict single-cell response to drug treatment. We also added new results for the developing pancreas cells in Fig. S13, which shows that MichiGAN has excellent disentanglement and generation performance on this real biological dataset.*

Reviewer #2: The authors first assessed the disentanglement and the generation performance of GANs and VAEs on a simulated scRNA-seq dataset and proposed MichiGAN to combines the strengths of VAEs and GANs. They applied MichiGAN to simulated and two real scRNA-seq datasets, and showed the disentangled representations can capture the aspects of cellular identity and predict unseen combinations of cell states. Overall, I feel this method somewhat interesting, but still have quite a few critical concerns, which need to be fully addressed.

Major:

1. Why were exactly 10 latent dimensions adopted? What are the results for 4 latent dimensions, for there are only 4 ground-truth factors in the simulated data?

*By using more latent variables than ground-truth variables, we followed the convention of previous papers that evaluated disentanglement performance, including the FactorVAE and beta-TCVAE papers. This practice also emulates real-world applications, where the number of ground-truth factors of variation is unknown. Specifying a larger number of dimensions gives the flexibility to explore possible factors of variation and enables the model to collapse the remaining dimensions to noise if they are not needed.*

*We re-ran the evaluations on the two Splatter simulated datasets, learning 4 latent dimensions for each method instead of 10. In two new figures (Fig. S8 and Fig. S9), we present the disentanglement performance of PCA, VAE and beta-TCVAE for splatter-simulated data with non-linear and linear steps, respectively. The beta-TCVAE representations still show the best disentanglement performance. We also present the disentanglement metrics of the three methods with 4 latent dimensions in Table 2.*

*We have added the results of disentangled representation learning methods with four latent dimensions on the splatter-simulated datasets as a paragraph in the results subsection “Variational autoencoders learn disentangled representations of single-cell data”.*

2. Fig.1b clearly shows the disentangled property of features learned by PCA, the linear relationship is very straightforward through eyeballing. And the Spearman Correlation displayed in Fig.1c confirms this. The disentangled property seems better persevered by PCA than beat-TCVAE in linear measurement. The authors may use the PCA representation as the input of GAN as a baseline.

*A key advantage of our MichiGAN approach is that any latent representation, including that from PCA, can be used to train the conditional GAN. Thus, although our evaluation showed that beta-TCVAE has the best disentanglement performance, any other dimensionality reduction approach could be used to train MichiGAN instead. Following your suggestion, we have trained MichiGAN on the simulated datasets using principal components as a baseline model instead of the latent variables from beta-TCVAE (Fig. S3). As expected, MichiGAN-PCA preserves the disentanglement performance of PCA. In Table 4, we also show the Spearman correlation gap of PCA and MichiGAN-PCA over 5 runs. The MichiGAN-PCA framework has very similar disentanglement metric values to those of PCA.*

*While PCA performs well at disentangling the linear ground-truth variables in our splatter-simulated datasets, it still has overall inferior disentanglement performance compared to the VAE methods and does not effectively disentangle nonlinear factors of variation. We have also used the PROSSTT package to simulate three new datasets (see below for details). We found that PCA performs significantly worse than VAE and beta-TCVAE at disentangling branch, time, and library size in these datasets (Fig. S10-S12).*

*Fig. S10 shows results for one of the PROSST simulations. Unlike the results for splatter-simulated data, which have some ground-truth variables that are linearly related to the original data, most of the PCA representations show only moderate mutual information with each ground-truth variable. Similar results for two other simulated datasets are shown in Fig. S11 and Fig. S12. We also observed similar results for the real dataset of developing pancreas cells that we added during revision.*

3. The conclusions about GANs and VAEs were made by comparing their performances on only one simulated dataset, which is not sufficient for validation. And only two real data were tested.

*In addition to the two types of simulations we performed previously, we have added three additional simulated datasets generated from the PROSSTT package [1], which simulates cell trajectories (developmental processes). We show disentanglement results for three PROSSTT-simulated datasets with 3, 4, and 5 trajectories, respectively, in Fig. S10-S12. We consider ground-truth variables of branch, time quartile, and library size quartile and show their mutual information to the latent variables obtained from PCA, VAE and beta-TCVAE in Fig. S10-S12. In addition, we also show disentanglement metrics for the three methods on the three PROSSTT-simulated datasets in Table 3. The beta-TCVAE method still performs the best among the three approaches by a wide margin. We have summarized these results in a paragraph in the results subsection “Variational autoencoders learn disentangled representations of single-cell data”.*

*In addition, we have added an additional real dataset from developing pancreas [2] in Fig. S13. We evaluated the disentanglement performance of PCA, VAE and beta-TCVAE on this dataset. Fig S13c shows that beta-TCVAE achieves significantly better disentanglement performance on this real dataset than PCA and VAE, and MichiGAN significantly improves the generation performance of beta-TCAE.*

*We have added the results of disentanglement and generation performance of different methods on the developing pancreas data as a paragraph in the results subsection “MichiGAN samples from disentangled representations without sacrificing generation performance”.*

*References:*

*Papadopoulos, N., Gonzalo, P. R., & Söding, J. (2019). PROSSTT: probabilistic simulation of single-cell RNA-seq data for complex differentiation processes. Bioinformatics, 35(18), 3517-3519.*

*Bastidas-Ponce, A., Tritschler, S., Dony, L., Scheibner, K., Tarquis-Medina, M., Salinno, C., ... & Bakhti, M. (2019). Comprehensive single cell mRNA profiling reveals a detailed roadmap for pancreatic endocrinogenesis. Development, 146(12).*

4. Besides, the observations and rules on the simulated data may not hold when it comes to real scRNA-seq datasets. And how do other methods (like WGAN and beta-VAE) perform on the real datasets you used for the case-study?

*To demonstrate that our observations on the simulated data also hold for the real data, we analyzed an additional real dataset from developing pancreas cells. Using this dataset, we evaluated the disentanglement performance of PCA, VAE and beta-TCVAE (Fig. S13). We also compared the generation performance of all methods, including Gaussian mixture model (GMM), PCA, VAE, beta-TCVAE, WGAN-GP and MichiGAN. Fig S13c shows that beta-TCVAE achieves significantly better disentanglement performance than PCA and VAE, and MichiGAN significantly improves the generation performance of the VAE models. Thus, both simulated and real datasets confirm the advantages of MichiGAN in terms of disentanglement and generation performance.*

*We have also added latent traversal plots using WGAN-GP and MichiGAN for the sci-Plex dataset (real data) to Fig. 5 and Fig. S6 and added the WGAN-GP latent traversals to Fig. 5.*

*In addition, following your suggestion, we have compared VAE and MichiGAN with VAE on the drug prediction task (Fig. S15). MichiGAN with VAE significantly outperforms VAE at predicting different cell type-treatment combinations using latent space vector arithmetic (Fig. S15b).*

*We have added the results of VAE and MichiGAN with VAE on the sci-Plex data to predict responses to drug treatments as a paragraph in the results subsection “Accuracy of latent space arithmetic influences MichiGAN prediction accuracy”.*

*It is not possible to perform the drug prediction task with WGAN-GP, so we cannot directly compare it to MichiGAN on this task. However, we have compared WGAN-GP and MichiGAN in terms of data generation performance on real datasets, including the tabula muris and pancreatic endocrinogenesis datasets.*

5. How do the other traditional methods other than GANs or VAEs perform in your cases? Is it necessary to apply a quite complicated deep generative neural network to scRNA-seq datasets, which usually have only a limited number of very tight cell clusters? What is the point that other simpler methods could not achieve?

*We have added the generation performance of two traditional methods--PCA and Gaussian mixture model (GMM)--on the Tabula Muris dataset. We showed their generation performance in Fig. 2. Those traditional methods have significantly worse data generation performance than VAE and WGAN-GP.*

*We think the reason that the traditional methods do not achieve better results than deep generative models is that there is more biological variation than simply cell clusters. For example, developmental time is an important underlying variable in the pancreatic endocrinologist dataset, and the type of effects exerted by different drug families is an important underlying variable in the sci-Plex dataset.*

*Furthermore, PCA achieves worse disentanglement than the VAE-based methods, as discussed above, and GMM does not provide any sort of latent representation.*

*In summary, deep generative models are powerful tools to disentangle complex factors of cellular identity and generate new samples of cellular expression profiles, with significant advantages over traditional approaches.*

6. The authors show the Random Forest Error of VAE, beta-TCBAE, WGAN-GP, MichiGAN in Fig.4. How robustness of this result? The robustness of MichiGAN should be explored since it is a two-stage method.

*To assess the robustness of our data generation and disentanglement results, we have re-trained each model 5 times. In Table 1 (disentanglement performance of PCA, VAE and beta-TCVAE), we show the mean and standard deviation of the metrics on each dataset. The beta-TCVAE still consistently has the highest values for FactorVAE and mutual information gap metrics.*

*In addition, we show the mean generation metrics along with their standard deviations as error bars in Fig. 2. The GAN methods robustly outperform the VAE-based methods, giving higher random forest error and inception score.*

*We also assessed the robustness of MichiGAN by re-training 5 times (Fig. 4c-d). Because MichiGAN is a two-stage process, as you noted, we performed end-to-end training (including beta-TCVAE and PCWGAN-GP) 5 times. Thus, the error bars show the overall robustness of the two-stage MichiGAN training process. MichiGAN shows larger variability than WGAN-GP, but still clearly outperforms the VAE approaches.*

7. Moreover, since the authors use different β values for different experiments, how to select this hyperparameter in real applications?

*Because β is a hyperparameter in an unsupervised learning approach (no ground truth is available in general), there is no direct way to pick a single best value for β. This is not a problem unique to the beta-TCVAE, but is a general challenge with any unsupervised learning approach. Our best recommendation is to choose a value in the range of 10-50 and use whatever biological prior knowledge is available, such as annotations of cell time point, condition, or cell type, to qualitatively assess the disentanglement of representations for different βvalues. One of the best things one can hope for with unsupervised learning algorithms is that the results are robust to different hyperparameter settings. To show that this is true in this case, we measured disentanglement performance of VAE and beta-TCVAE for beta = 10 and 50 on the simulated datasets (Fig. S14). We found that beta-TCVAE with either β=10 or β=50gives a higher mutual information gap (MIG) than VAE in all cases. In short, even if you do not choose the perfect value of β, it is still better to use beta-TCVAE than VAE.*

*We have added this instruction in a new methods subsection “Tuning beta values in beta-TCVAE”.*

8. The authors demonstrated single-cell gene expression could predict unseen drug or drug combinations by MichiGAN. It seems this could be done in a simple way. Also the effectiveness should be compared with other related ones (e.g., The RF Error of WGAN-GP).

*We may not have clearly explained the task of generating single-cell gene expression changes under different drug treatments. Instead of a regression or classification task, the goal is to generate cell samples under different cell type/treatment combinations. Although traditional methods such as random forests, support vector machines, regularized linear regression, or feedforward neural networks can predict mean expression profiles through regression, they are unable to generate realistic samples from the distribution of possible single-cell expression profiles. On the other hand, deep generative models are able to model complex, high-dimensional data distributions and generate realistic samples. Because these traditional methods cannot be used to perform such sampling, we employed deep generative models instead. We are not aware of any way to use traditional methods for this task.*

*The WGAN-GP does not have an encoder to predict the latent subspace of cells of a certain drug/cell type combination, which makes it unable to perform latent space vector arithmetic. Thus, WGAN-GP cannot be directly used for the drug response prediction task at all, and we have no way to compare its performance with MichiGAN on this task. However, we did confirm that MichiGAN achieves comparable data generation performance to WGAN on real data. Furthermore, we have added the results of using VAE alone, as well as MichiGAN with VAE, for the drug prediction task (Fig. S15). MichiGAN with VAE shows significantly better drug prediction performance than VAE alone.*

Minor:

9. The authors may consider to put some technical details in the method description to make the result section easy to follow.

*Thanks for your suggestion. We have moved the technical details of Algorithm 1 to the method section to make the result section easier to follow.*

**Second round of review**

**Reviewer 2**

The authors have addressed my concerns.
